# Omics and imaging combinatorial approach reveals butyrate-induced inflammatory effects in the zebrafish gut

**DOI:** 10.1186/s42523-023-00230-2

**Published:** 2023-03-03

**Authors:** Adrià López Nadal, Jos Boekhorst, Carolien Lute, Frank van den Berg, Michelle A. Schorn, Tommy Bergen Eriksen, David Peggs, Charles McGurk, Detmer Sipkema, Michiel Kleerebezem, Geert F. Wiegertjes, Sylvia Brugman

**Affiliations:** 1 Cell Biology and Immunology Group, Wageningen University and Research, Wageningen, The Netherlands; 2 Aquaculture and Fisheries Group, Wageningen University and Research, Wageningen, The Netherlands; 3 Host-Microbe Interactomics, Wageningen University and Research, De Elst 1, 6708 WD Wageningen, The Netherlands; 4 Laboratory of Microbiology, Wageningen University and Research, Wageningen, The Netherlands; 5 Skretting Aquaculture Innovation, Stavanger, Norway

**Keywords:** Microbiome, Transcriptome, Omics, Imaging, Zebrafish, Butyrate, Soy saponin, Gut, Inflammation

## Abstract

**Background:**

Prebiotic feed additives aim to improve gut health by influencing the microbiota and the gut barrier. Most studies on feed additives concentrate on one or two (monodisciplinary) outcome parameters, such as immunity, growth, microbiota or intestinal architecture. A combinatorial and comprehensive approach to disclose the complex and multifaceted effects of feed additives is needed to understand their underlying mechanisms before making health benefit claims. Here, we used juvenile zebrafish as a model species to study effects of feed additives by integrating gut microbiota composition data and host gut transcriptomics with high-throughput quantitative histological analysis. Zebrafish received either control, sodium butyrate or saponin-supplemented feed. Butyrate-derived components such as butyric acid or sodium butyrate have been widely used in animal feeds due to their immunostimulant properties, thereby supporting intestinal health. Soy saponin is an antinutritional factor from soybean meal that promotes inflammation due to its amphipathic nature.

**Results:**

We observed distinct microbial profiles associated with each diet, discovering that butyrate (and saponin to a lesser extent) affected gut microbial composition by reducing the degree of community-structure (co-occurrence network analysis) compared to controls. Analogously, butyrate and saponin supplementation impacted the transcription of numerous canonical pathways compared to control-fed fish. For example, both butyrate and saponin increased the expression of genes associated with immune response and inflammatory response, as well as oxidoreductase activity, compared to controls. Furthermore, butyrate decreased the expression of genes associated with histone modification, mitotic processes and G-coupled receptor activity. High-throughput quantitative histological analysis depicted an increase of eosinophils and rodlet cells in the gut tissue of fish receiving butyrate after one week of feeding and a depletion of mucus-producing cells after 3 weeks of feeding this diet. Combination of all datasets indicated that in juvenile zebrafish, butyrate supplementation increases the immune and the inflammatory response to a greater extent than the established inflammation-inducing anti-nutritional factor saponin. Such comprehensive analysis was supplemented by in vivo imaging of neutrophil and macrophage transgenic reporter zebrafish (mpeg1:mCherry/mpx:eGFPi^114^) larvae. Upon exposure to butyrate and saponin, these larvae displayed a dose-dependent increase of neutrophils and macrophages in the gut area.

**Conclusion:**

The omics and imaging combinatorial approach provided an integrated evaluation of the effect of butyrate on fish gut health and unraveled inflammatory-like features not previously reported that question the usage of butyrate supplementation to enhance fish gut health under basal conditions. The zebrafish model, due to its unique advantages, provides researchers with an invaluable tool to investigate effects of feed components on fish gut health throughout life.

**Supplementary Information:**

The online version contains supplementary material available at 10.1186/s42523-023-00230-2.

## Background

In the last decades, the implications of the microbiome in human and animal health have gained interest among and beyond the scientific community. As a consequence, food ingredients able to modulate the microbiome, such as prebiotics, became increasingly popular and accepted among the general public and have been utilized in human dietary supplements as well as in animal feed [17, 39]. A prebiotic is a substrate that is selectively utilized by host microorganisms and thereby proposed to confer a health benefit on the host (reviewed in [26].

Butyrate is a short-chain fatty acid (SCFA) derived from fiber fermentation by the gut bacteria that exhibits some prebiotic properties, playing a role in the interaction between bacterial population dynamics and host gut homeostasis [44]. Butyrate has a direct impact on the immune system via signaling G-protein coupled receptors (GPCR) on epithelial and immune cells and also induces epigenetic changes via regulation of histone acetylase and histone deacetylase enzymes (reviewed in [31]. In the last years, butyrate has been extensively used in animal feed, including its supplementation to several fish diets in the form of butyric acid or sodium butyrate due to its growth-promoting, immuno-stimulating and antioxidative properties (reviewed in [1] and to mitigate detrimental effects of sub-optimal plant-containing diets [23, 48, 70, 83].

Plant-based protein ingredients have been replacing fish meal in feed due to their more favorable price and availability. However, several anti-nutritional components derived from plant-based protein sources are reported detrimental for fish health (reviewed in [73]. For instance, soy saponin is an anti-nutritional component of soybean meal that interacts with cell membranes and promotes pore formation, vesiculation and membrane domain disruption [4]. Various studies linked the presence of soy saponin to inflammatory responses in the intestinal mucosa, enteritis as well as microbiota modulation in several fish species [13, 16, 40], including zebrafish [49]. In zebrafish larvae, the number of neutrophils increased in the gut after soybean meal feeding [30] or exposure to soy saponin in solution [49]. After assessing the inflammatory effect of soy saponin, soy-containing diets have been employed as a model for feed-induced inflammation to decipher the underlying diet-microbe-host interactions in the zebrafish gut and to assess feed compounds that can potentially protect the gut from becoming inflamed [77]. Experimental designs of fish feed studies are often based on specific outcome parameters, including fish growth, expression of a limited set of genes, plasma levels of antioxidants, semi-quantitative scoring of histological parameters, profiling of the gut bacteria composition or pathogenic challenges. Habitually, only end-point analysis have been performed, ignoring the kinetics of the responses. Most of these studies base eventual gut health claims of dietary treatments on one or two of these parameters. For instance, the anti-inflammatory properties of supplemented microalgae were assessed by only quantifying the neutrophils in the zebrafish gut larvae [10]. The expression of a small set of genes were proposed to support immune-boosting, anti-inflammatory and antioxidative stress properties of phytates after soybean-meal feeding in zebrafish larvae [74]. Although quantitative assessment of health associated phenotypes is critical to support health claims, basing these on the determination of a single or only few parameters may lead to overstated conclusions. Therefore, to appropriately assess and understand the complex and multifaceted effects of feed supplements on fish gut health more integrated and holistic approaches are warranted (as reviewed in [50]). In order to achieve this, we propose that a combination of high-throughput readouts may be employed to supply unbiased datasets that could lead to a detailed description of the host gut health status upon feed interventions.

Many gut functions and immune genes are conserved between different species. Moreover, zebrafish larvae are optically transparent and together with the development of several transgenic fish lines that express fluorescent proteins in specific cell-lineages facilitates in vivo tracking of certain immune cells, which empowered the use of the zebrafish model to examine intestinal inflammation (reviewed in [12] as well as a model organism to evaluate novel feeds for farmed fish [79]. However, the zebrafish gastrointestinal tract presents several particularities. For example, zebrafish lack a stomach and instead employ the anterior gut segment, named intestinal bulb, as a reservoir for feed. Although this intestinal bulb lacks gastric glands, it produces digestive enzymes and mimics what may occur in the stomach [22, 57]. The zebrafish gut epithelial layer also lacks intestinal crypts that are typically found in other fish species or in mammals and rather forms protrusions called folds that decrease in size from anterior to posterior gut segments [85]. Nevertheless, the canonical intestinal epithelial cells (IECs) such as enterocytes, mucin-producing goblet cells and enteroendocrine cells are present in the zebrafish gut. Moreover, zebrafish gut segments presented analogous expression to their mammalian counterparts [87] and when transcriptomics where performed on IECs from zebrafish, stickleback, mouse and human a highly conserved expression was found between zebrafish and mammals [46]. Like in many animal models for inflammation, mucus-producing cells (Goblet cells) decrease and granulocytes (mainly neutrophils and eosinophils), macrophages and lymphocytes increase upon inflammation in the zebrafish gut [11, 49]. Due to its shared expression and functionality the zebrafish is an excellent model to understand host-microbe-immune interactions.Research on feed supplements and their effect on gut health are usually based on few readouts parameters which may not reflect the complexity of fish gut health. The main goal of this present study is to provide an comprehensive investigation based on the integration of several high-throughput readouts of the gut mucosa to depict a more holistic view of the effects of feed supplements on zebrafish gut health.

## Material and methods

### Ethics statement

The present study was approved by the Dutch Committee on Animal Welfare (2017.W-0034) and the Animal Welfare Body (IvD) of the Wageningen University (The Netherlands). Furthermore, we adhered to standard biosecurity and institutional safety procedures at Wageningen University and Research.

### Zebrafish and diets

Adult double transgenic (mpeg1:mCherry/mpx:eGFPi^114^) expressing mCherry under the macrophage-specific mpeg1 promotor and GFP under the neutrophil-specific mpx promotor fish were housed and fed as previously described [49]. Embryos were obtained by natural spawning. Fish were fed as follows: weeks 1 and 2 with rotifers (× 4/day from 5 days post fertilization -dpf-), week 3 with rotifers and Artemia Nauplii 230.000 npg (Nauplii per gram) (Ocean Nutrition Europe, Essen, Belgium) (× 2/day), week 4 with Artemia (× 2/day) and until 40 dpf Artemia and Tetramin Flakes (Tetra, Melle, Germany) (× 2/day). When fish reached the juvenile stage, at 40 dpf [76], fish were randomly distributed into 6 tanks, 2 per each diet: one was sampled after 1 week and the other tank after 3 weeks of the feeding experiment per each diet. The feeding experiment was performed blind and fish were fed until slightly before satiation twice a day. Each tank received one of the following: a control diet, a saponin-supplemented diet or a butyrate-supplemented diet. Full diet composition is listed in Table 1.

**Table 1:** Formulation of experimental diets analysed once the feed intervention was performed to check which compositions corresponded to the blinded diets

	A: Control diet (%)	B: Butyrate diet (%)	C: Saponin diet (%)
Wheat	7.00	6.99	6.67
Wheat gluten	16.00	16.00	16.00
Sunflower meal	1.68	1.68	1.68
Soy protein concentrate	15.16	15.16	15.16
Fish meal	52.00	52.00	52.00
Fish oil	4.40	4.40	4.40
Rapeseed oil	2.00	2.00	2.00
Vitamin mix	0.35	0.35	0.35
Mineral mix	1.92	1.92	1.92
Butyrate	0.00	0.01	0.00
Saponin	0.00	0.00	0.33
[VOLUME]	100.0	100.0	100.0
Dry matter	92.2	92.0	92.0
Crude protein	56.0	57.4	57.4
Crude fat	13.5	13.8	13.8
Ash	8.9	8.9	8.9

The three diets are similar in composition (dry-matter, protein, fat and ash). 95% ultrapure soy saponin was kindly provided by Trond Kortner NMBU Oslo Norway, origin: Organic Technologies, Coshocton, OH, [40]

### Experimental design

**Fig. 1:**
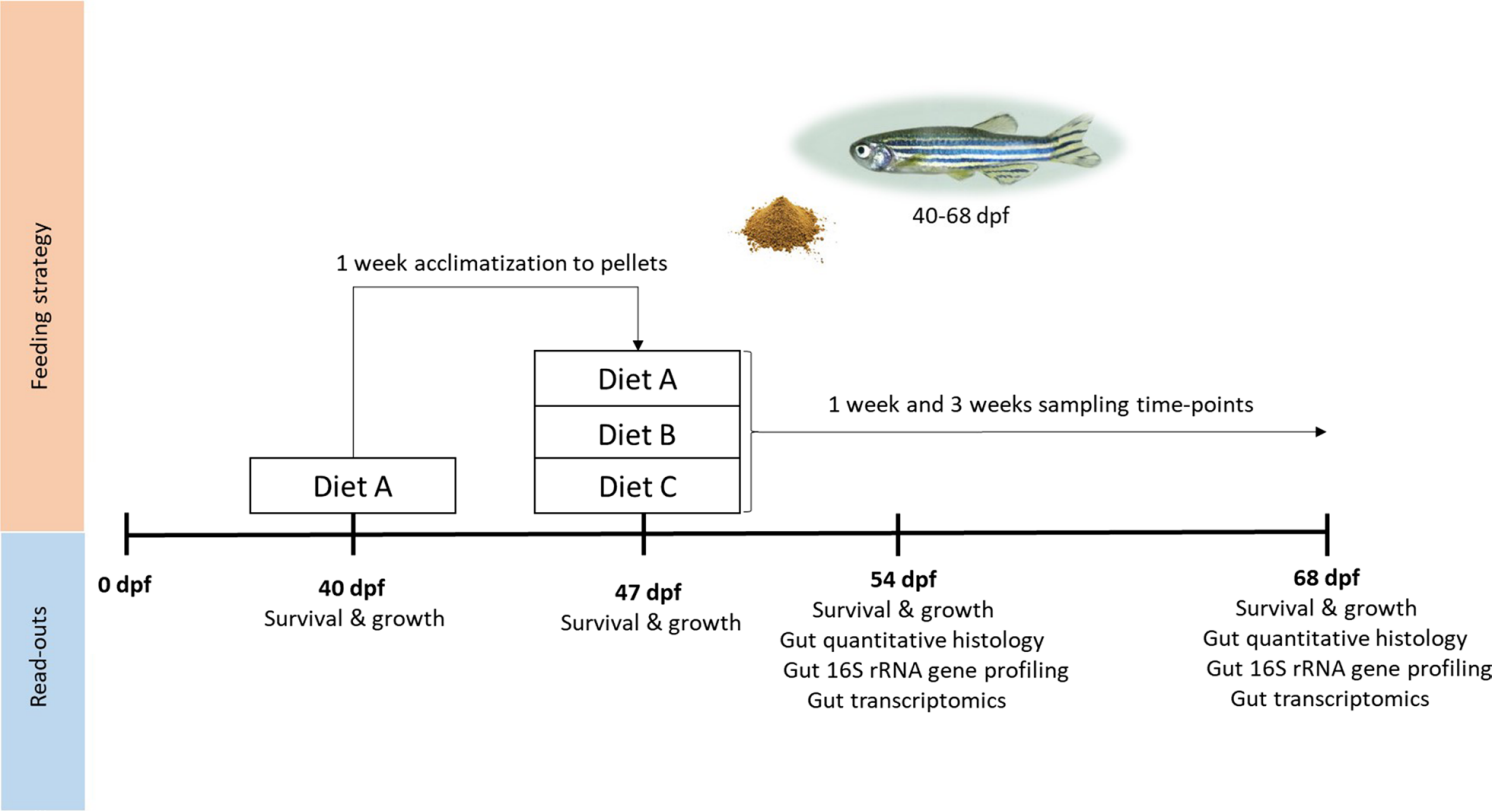
Experimental design. Fish were bred and raised as described in the section ‘Zebrafish and diets’. At 40 dpf (juvenile stage) they were fed diet A for 1 week for acclimatisation to dry feed pellets. At 47 dpf, fish were randomly distributed into tanks and fed one of the diets (A, B or C) for 3 weeks. Survival and growth were measured before and during the whole experiment. After 1 week (54 dpf) and after 3 weeks (68 dpf) of feeding the fish. Gut samples were collected for histological, metatranscriptomic and microbiome analyses

Water quality was set to standard values by replacing half of the water in the zebrafish system before the start of the experiment and monitored twice a week during the whole experiment (Additional file 1: Fig. S1). A pH meter (Hanna Instruments, Nieuwegein, The Netherlands) was used to measure the pH and the water conductivity. Kits to measure ammonium, nitrite and nitrate (Merck KGaA, Darmstadt, Germany) were used according to manufacturer’s instructions. Additionally, nitrite, nitrate, general hardness, carbonate hardness, pH and chlorine were (re)measured by using Tetra Test 6in1 (Tetra, Melle, Germany) according to manufacturer’s instructions. Fish survival and standard length -from the tip of the head until the bifurcation of the caudal fin- were assessed by using a digital caliper (Sylvac, Yverdon, Switzerland) during the experiment (Additional file 2: Fig. S2). The dietary intervention consisted of three diets identical in composition except the supplementation with 1 g/kg feed of sodium butyrate in the butyrate diet and 3.3 g/kg of 95% ultrapure soy saponin in the saponin diet (Table 1). We sampled fish guts after 1 week (54 dpf, 1^st^ timepoint) and after 3 weeks (68dpf, 2nd timepoint) after the start of the dietary intervention. Fish were fed twice daily until satiation and the amount of feed provided was quantified with a micro-spoon, feeding 15.1 mg of feed per tank per day (averaging to 0.46 mg of feed per day per fish). A summary of the experiment design is depicted in Fig. 1.

### Single gut and water samples RNA extraction

**Fig. 2:**
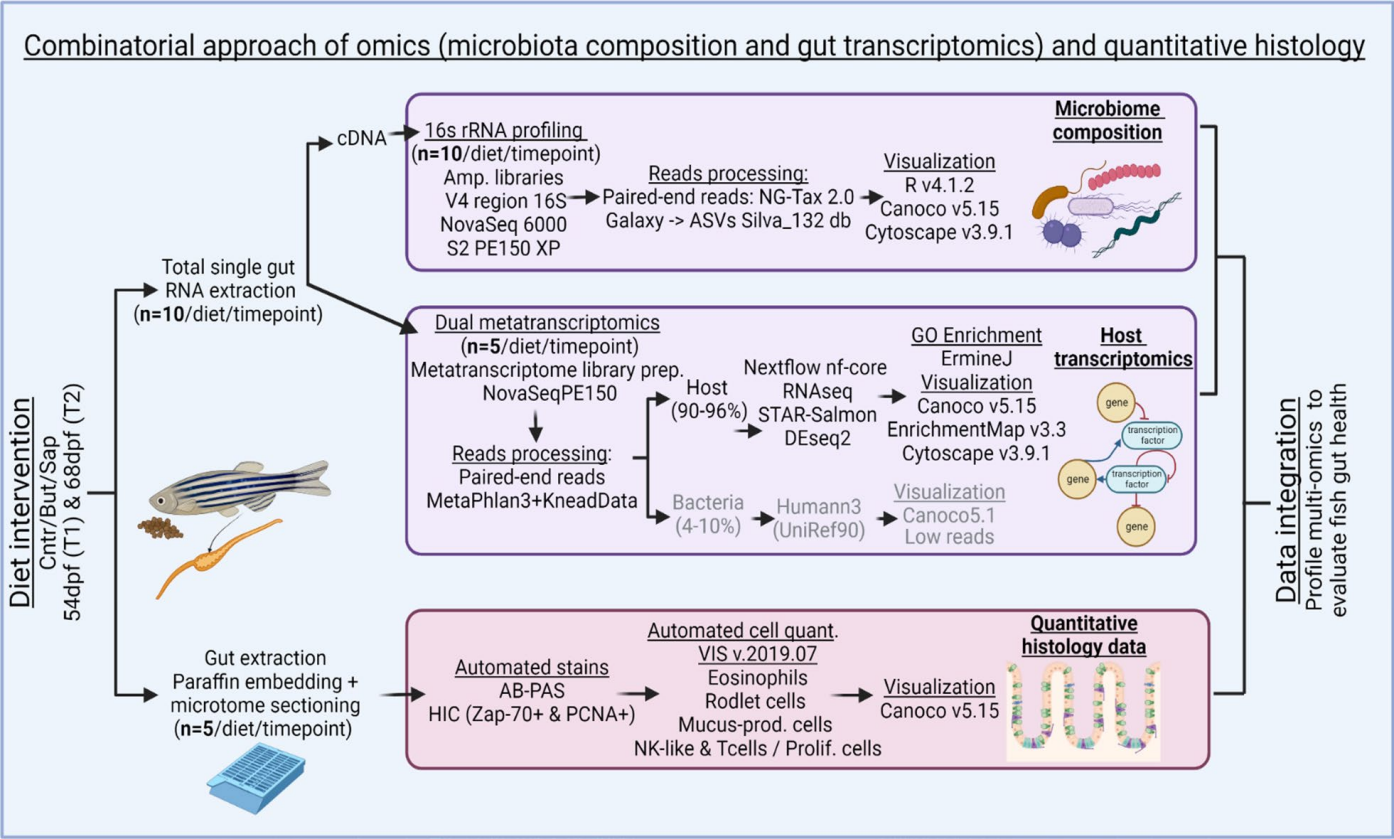
Combinatorial approach employed: total RNA was extracted from single zebrafish gut fed on different diets for both timepoints. Aliquots of total RNA were used for cDNA. For the 16S rRNA gene profiling, amplicon libraries of the V4 region of the 16S RNA gene were generated from the cDNA synthetized. NG-Tax 2.0 Galaxy was sued to obtain the ASVs. Several packages of R v4.1.2., Canoco v5.15 and Cytoscape v3.9.1 were used for results visualization. For transcriptomics, the cDNA libraries were sent to NovaSeq 6000 PE150 for sequencing. MetaPhlAn 3.0 (Beghini et al. 2021) and KneadData were used to trim the overrepresented sequences. Nf-core/rnaseq Nextflow pipeline was used for processing of the reads with the GRCz11 genome assembly. The results were visualized by R v.4.1.2, Canoco v5.15 and ErmineJ was used for the GO Enrichment analysis. The histological samples were extracted and embedded in paraffin and sectioned using a microtome. AB-PAS and HIC stains were automated. Samples were digitally scanned and an automated quantification of the histological parameters was performed using VIS v.2019.07 and Canoco v5.15 and GraphPad Prism v9.0.0 to visualize the results. The data integration was performed using heatmaps of normalized relevant parameters from all datasets, both timepoints and all diets

Guts were extracted from juvenile zebrafish, rinsed in sterile PBS, snap frozen in liquid nitrogen and preserved at − 80 °C for total gut RNA extraction. RNA extraction was performed from single intestines as previously described [35]. Water samples were obtained by filtering 2L of water from each fish tank using Nalgene™ Rapid-Flow™ Sterile Disposable Bottle Top Filters with PES Membrane 0.45 µm (ThermoFisher Scientific, MA, USA). Aliquots of total RNA were used for cDNA synthesis with the Maxima H minus First Strand cDNA Synthesis Kit (ThermoFisher Scientific, MA, USA), following the standard protocol using random hexamer primers to create cDNA. This cDNA was used for 16S rRNA gene profiling of the bacterial communities and the extracted RNA was used for metatranscriptomic analysis. The quantity, quality and purity of total RNA was determined using the Qsep100™ Bio-Fragment Analyzer (Bioptic inc., New Taipei City, Taiwan) and the Qubit™ RNA BR Assay Kit (ThermoFisher Scientific, MA, USA). A schematic pipeline of the whole process from sample collection to results analyses is depicted in Fig. 2.

### Microbiome: 16S rRNA profiling and sequencing data analysis

Samples from juvenile zebrafish fed the three different diets were sampled from individual separate tanks. Amplicon libraries of the V4 region of the 16S rRNA were generated from the cDNA synthetized from single gut and water samples, using barcoded and modified F515-806R primers [86]. The PCRs were performed in triplicate, purified, and quantified as previously described [29]. Purified PCR amplicons were pooled in an equimolar mix and sent for library preparation and sequencing using the Illumina NovaSeq 6000 S2 PE150 XP technology at Eurofins Genomics Germany GmbH (Eurofins Genomic, Ebersberg, Germany). Raw paired-end reads were analyzed using the standard parameters of NG-Tax 2.0 [66], with the exception of using 100 bp as the forward and reverse read length, as implemented in Galaxy [2], to obtain Amplicon Sequence Variants (ASVs). Taxonomy was assigned to ASVs using the Silva_132 database [67]. Two synthetic “mock communities” with known compositions were amplified and sequenced as positive controls and a no-template control was also included as a negative control [68]. The distribution of reads per sample and the variance in ASVs were assessed and Alpha- and Beta-diversity measurements were performed using R v4.1.2 and RStudio [43], using packages ggplot2, [89], ape, [61], plyr, [93], vegan, [59], RColorBrewer, [58], reshape2, [90], scales [91], data.table, [19], microbiome, [42], dplyr, [92], phyloseq, [55], ggdendro, [84] and DT [95]. The analysis yielded 17,203,234 high-quality reads. We excluded one sample (54 dpf butyrate diet) because it had 2 reads only and we kept all the other samples (> 30.000 reads). Rarefaction curves for all samples reached a plateau, indicating that sufficient sequencing depths was achieved (data not shown). For the calculation of alpha-diversity indices, data was rarefied against the sample containing the lowest number of reads (31,814 reads). Redundancy analysis (RDA) and principal component analysis (PCA) were performed with Canoco v5.15 [9] using analysis type “constrained” or “unconstrained”, respectively. Response variables were log-transformed with the formula log(10,000*relative_abundance + 1). RDA *p*-values were determined through permutation testing (500 permutations). Boxplots were generated using Prism v.9.0.0 (GraphPad Software, San Diego, California USA). Cytoscape v3.9.1 [75] was used to visualize the diet-specific co-occurrence of ASVs based on their relative abundances. Additional data handling and format conversions were done in Python (https://www.python.org/).

### Zebrafish gut transcriptome analyses

Total RNA (n = 5 diet/timepoint) was sent to Novogene (Cambridge, UK), where quality control was done, rRNA was depleted and the metatranscriptome libraries were prepared. Paired-end reads were generated by NovaSeq 6000 PE150. For the host reads we used nf-core/rnaseq Nextflow pipeline [21] and the zebrafish (*Danio rerio*) genome assembly GRCz11 (NCBI) and according to the MultiQC reports generated, the quality check parameters were satisfactory for all samples. “Salmon” was used to quantify the expression of the transcripts [62] and DEseq2 [52], ggplot2, [89], scales [91], viridis [24] in RStudio to investigate the differentially expressed genes (DEG) in our diet treatments and timepoints. PCA analyses were performed in the Canoco v5.15 software suite (v5.02, [9]. Gene Score Resampling (GSR) analyses was performed ErmineJ (v3.1.2) [27] with the annotation file of zebrafish (*Danio rerio*; genome assembly GRCz11) generated by Gemma [97]. GSR used DEG scores from DEseq2 from all genes in the dataset and calculated a *p*-value for each Gene Ontology (GO) term. The fold-change of each GO term across dietary interventions was calculated by collapsing individual transcripts per million (tpm) of each gene to the belonging GO term(s). Differentially expressed GO terms were visualized as a network by using Cytoscape v3.9.1 [75]: the nodes contained the fill depicting the log2 fold change (FC) of the control vs butyrate at T2 and the border depicting the log2 FC of the control vs saponin at T2. The nodes with an absolute FC ≥ 0.5 and *p* value ≤ 0.1 between dietary interventions were taken into account. The edges connected the relevant nodes if the GO term contains at least 10 genes and shared at least half of them with the connecting GO term(s) with FC ≥ 0.2 and *p* value ≤ 0.05 between dietary interventions. All data and files used to generate these visualisations can be found in Additional file 9.

### High-throughput quantitative histology

At 54 dpf and 68 dpf zebrafish were euthanized in buffered MS222 overdose [88] 250 mg/L Tricaine (Sigma-Aldrich, DL, United States). Intestines were removed, rinsed in PBS, placed in 4% paraformaldehyde overnight and transferred to 70% ethanol on the next day. After subsequent dehydration steps, total intestines were embedded in paraffin blocks. Five-micrometer sections were stained with one of the following: hematoxylin and eosin (H&E) or Alcian blue periodic acid-Schiff (ABPAS) as previously described in [11] or by immunohistochemistry (IHC). For the latter, antigen retrieval was performed using the PT Link automatic antigen retrieval machine (Dako Agilent, CA, USA): samples were placed into citrate buffer pH 6.1 (Dako Agilent, CA, USA) at 60 °C, heated to 97 °C in 20 min, kept at 97 °C for 20 min, and cooled down to 60 in 20 min. Samples were stained using an automated staining machine (Autostainer Link 48, Dako Agilent, CA, USA) with anti-proliferating cell nuclear antigen (PCNA mouse mAb Clone PC10, M0879, Dako A/S, Denmark, diluted 1:10.000) or with anti-Zeta chain of T cell receptor associated protein kinase 70 (ZAP70 Rabbit mAb 99F2, Cell Signaling Technology USA, diluted 1:300) antibodies to study proliferating cells (epithelial renewal) as well as NK-like cells and T cells. Samples were scanned at 20× magnification using Pannoramic SCAN II (3DHISTECH, Budapest, Hungary) to produce digital whole slide images and analyzed using Visiopharm v. 2019.07 image analysis software (Visiopharm, Hoersholm, Denmark). Specialized automated image analysis protocols were developed for each staining type. Before employing the quantitative histology, tissue regions were manually defined on the images to select representative tissue (avoiding artefacts). The automated analysis was then preformed only within those regions. Making an automated protocol involves selecting pre-processing steps, such as median filters to reduce noise and enhance structures, training the Bayesian classifier algorithm by annotating examples of the image background, tissue, and target cells, utilizing post-processing steps based on shape, size and pixel colour to enhance the final image segmentation, and define calculations to give the output data (area, counts, and perimeters). This method for image analysis allowed us to perform quantitative histology which differs from the commonly used semi-quantitative scoring. The latter involves a pathologist ascribing a subjective scoring with ordinal data, which is strongly operator-biased and time consuming. Quantitative histology is automated, more detailed, thorough, and consistent, producing numerical data that can detect subtle differences between states. The cell types imaged were mucus (goblet) cells, PAS + cells (granulocytes), rodlet cells; PCNA + cells (proliferative cells) and Zap-70 cells (NK-like and T lymphocytes) [56]. The histological parameters quantified were as follows. Absorptive capacity (AC): which was formulated as (interface length between mucosa and lumen / interface length between serosa and exterior of the gut). Cell area fraction (%) (tissue area made up of cells) formula: area cell type A/total tissue area *100. Cell density: cell number/total tissue area. Cell size: area of cells/cell number. Cell distance: the distance of the cells from the outer serosal layer, where a higher distance would indicate a cell migrating towards the mucosal fold (villus) end towards the lumen. The imaging and the histological quantification were performed at the facilities of Skretting Aquaculture Innovation (Skretting, Stavanger, Norway). Further downstream processing of the multivariate analysis was performed by using Canoco v.5.12 (v5.02 [9], using principal coordinate (PCoA) and Redundancy (RDA) analyses, performing analysis type “unconstrained” and “constrained”, respectively. Response variables (histological parameters) were scaled (0–1) and biplots were generated. RDA *p*-values were determined through permutation testing (500 permutations). Boxplots were generated using Prism v.9.0.0 (GraphPad Software, San Diego, California USA).

### Complete pipeline of the combinatorial approach: omics and quantitative histology

#### *Fluorescent *in vivo* imaging experiment*

Adult Tg (mpeg1:mCherry/mpx:eGFPi^114^) were housed and fed as previously described [49]and embryos obtained by natural spawning and raised with E3 water (0.10 mM NaCl in demineralized water, pH 7.6) in petri dishes at 28 °C (12/12-h light/dark cycle) [88]. Larvae were randomly distributed in 6 well plates (*n* = 20 fish/well) and exposed to different concentrations [0.005, 0.01 mg/ml] of butyrate and [0.5, 0.7 mg/ml] saponin dissolved in E3 water (10 ml solution/well) from 3 to 6 dpf. Larvae were anaesthetized and in vivo imaged as previously described in [49]. Pictures were analyzed with ImageJ® software (United States National Institutes of Health, Bethesda, United States): the intestinal area was selected manually for each fish from the bright field and copied to the other channels and fluorescent cells quantified and boxplots were generated using Prism v.9.0.0 (GraphPad Prism Software, San Diego, California, USA).

## Results

### Butyrate and saponin diets did not affect survival nor fish growth

All of the fish survived the dietary intervention and fish growth was comparable regardless of the diet provided (Additional file 1: Fig. S1). The water quality indicators measured: water pH, water conductivity (µS/m), nitrite (NO_2_^−^ in mM), ammonia (NH_4_^+^ in mM), nitrate (NO_3_^−^ in mM), chlorine (Cl_2_^−^ in mM), general hardness (Ca^2+^ and Mg^2+^ per volume of water) and carbonate hardness (CaCO_3_ and MgCO_3_ per volume of water) were consistently within the recommended range (Additional file 2: Fig. S2). Moreover, water quality indicators remained constant during the whole experiment, indicating that the diet-related changes described below result from the dietary intervention and not from differences in fish growth rates or fluctuations in water quality.

### Butyrate- and saponin-supplemented diets altered gut microbiota composition over time

**Fig. 3:**
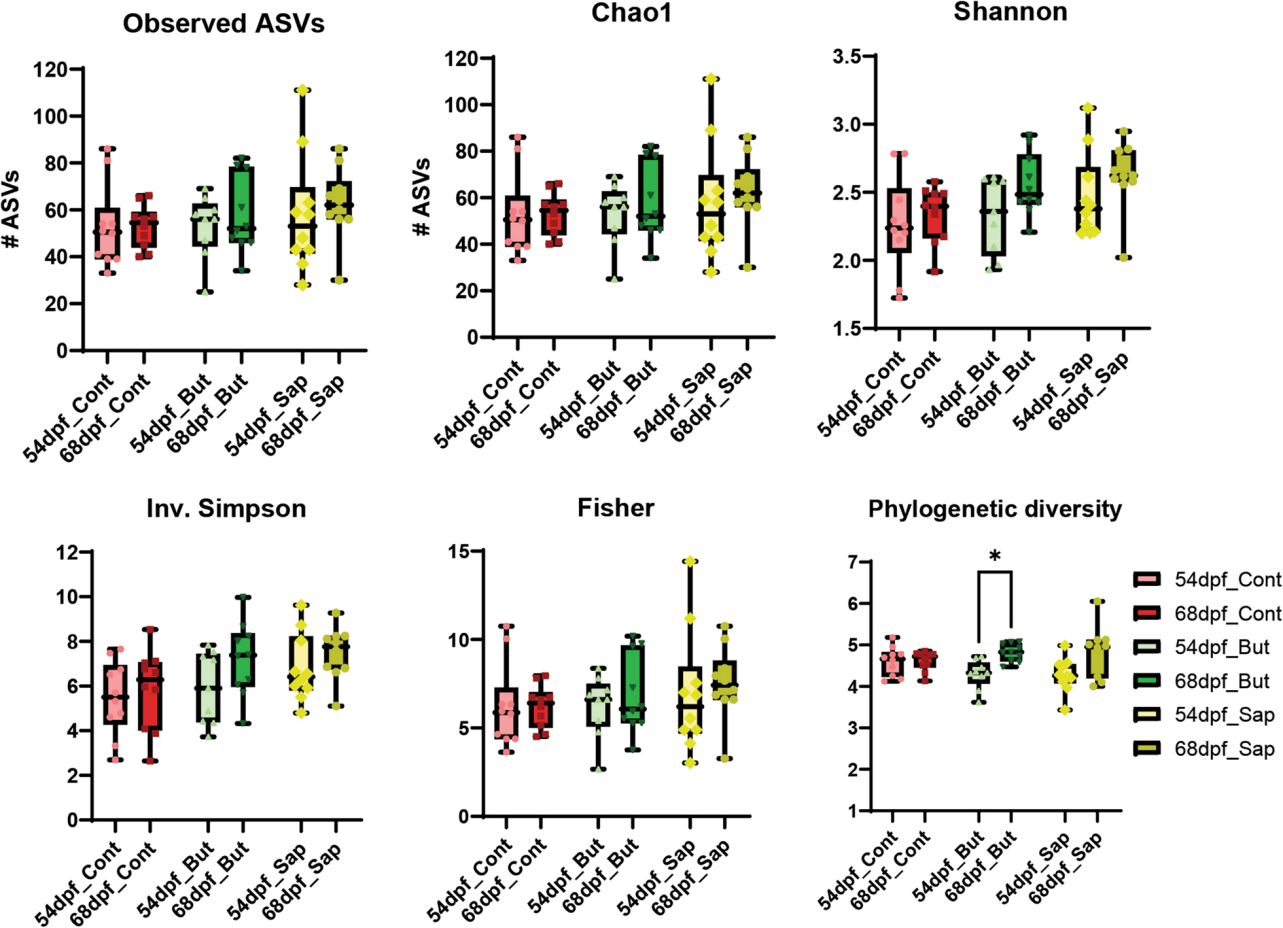
Alpha-diversity indexes for richness (observed ASVs and Chao1) and for diversity (Shannon, Inverse Simpson, Fisher and Phylogenetic Diversity). *p ≤ 0.05, Ordinary one-way ANOVA after confirming normally distributed data by Shapiro–Wilk test. Whiskers: min. to max. shall all points with median

Ten gut samples per diet per timepoint were used to determine prokaryotic community composition based on amplicon sequencing of 16S rRNA. The samples yielded 17,203,234 high-quality reads, with an average of 286,720 reads per sample, ranging from 31,814 to 577,719. The reads resulted in 579 amplicon sequence variants (ASVs) which were reduced to 204 ASVs after filtering out the ones occurring in ≤ 2 counts. Alpha-diversity indexes for richness (observed ASVs and Chao1) and diversity (Shannon, Inverse Simpson, Fisher and Phylogenetic Diversity) within the samples did not reveal any significant differences between diets and timepoints for all samples. Only the Phylogenetic Diversity slightly increased for butyrate fed fish overtime (Fig. 3).

**Fig. 4:**
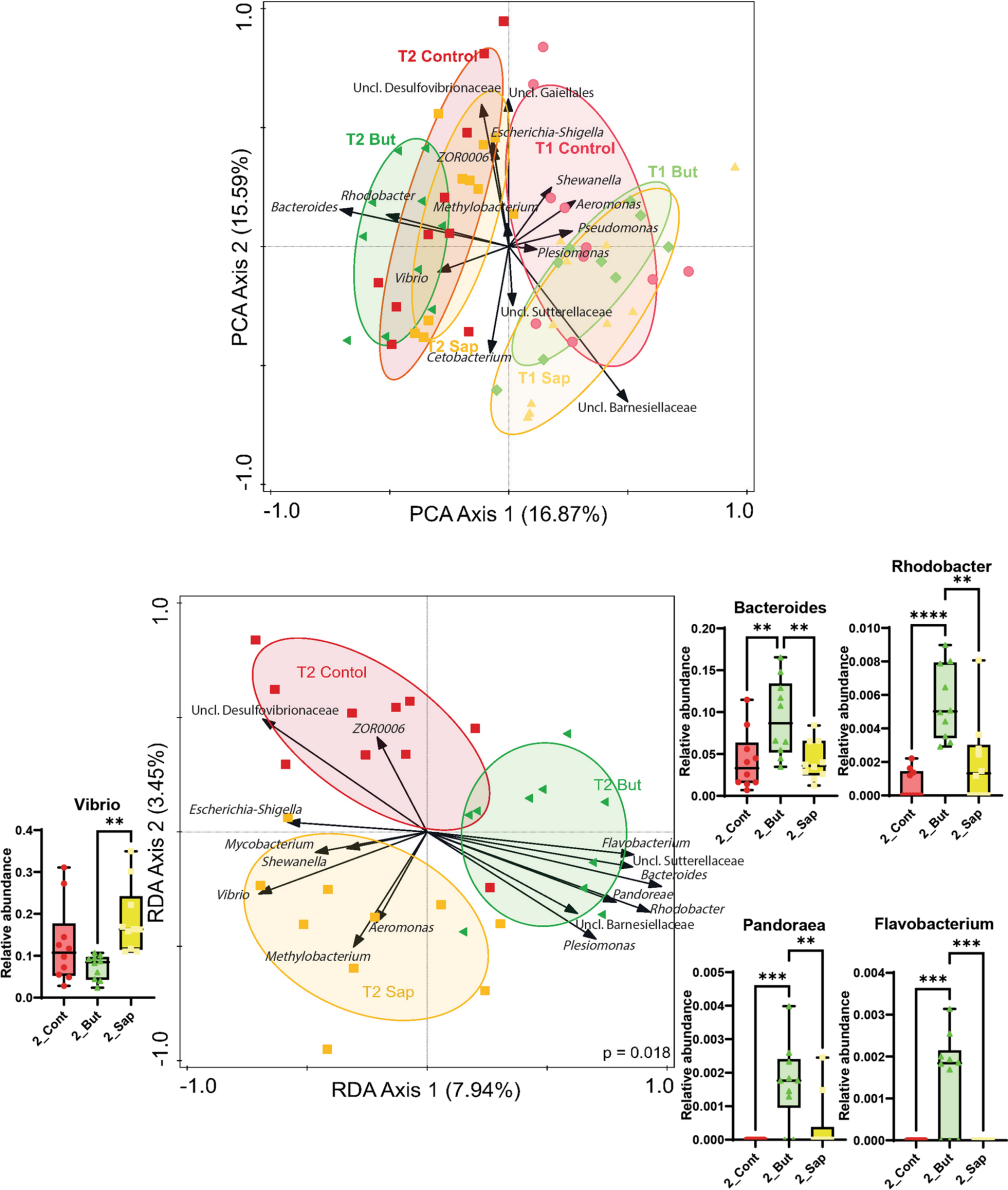
**A** Principal component analysis exploring the interaction of diet and time. The x axis separates the samples after 1 week feeding (54 dpf) from samples after 3 weeks feeding (68 dpf) and explains 16.87% of the variation observed. **B** Redundancy analysis of samples after 3 weeks of feeding (68 dpf), the x axis separates saponin from butyrate fed fish and explains 7.94% of the microbial differences observed and the y axis separates the control from the saponin fed fish and explains 3.45% of the microbial differences observed. The microbial communities changed significantly due to diets (*p* = 0.018). The relative abundance of the most discriminative genera are depicted with boxplots around the RDA. In both analyses, the top 15 most distinctive genera are represented with black arrows. The direction of the arrows correlated with the dietary treatments and the timepoints and their length correlate with the strength of the correlation. **p ≤ 0.01, *** *p* ≤ 0.005 one-way ANOVA test or Kruskal–Wallis test after testing for normality on data distribution by Shapiro–Wilk test. No false discovery rate performed. Whiskers: min. to max. shall all points with median

A principal component analysis (PCA) analysis shows that time (from 54 to 68 dpf) explains ~ 17% of the variation observed in the microbial communities (x-axis Fig. 4A). To analyze the effect of diet on the microbial communities, we performed redundancy analysis (RDA) separately for each timepoint. After one week on the different diets (54 dpf), the gut microbiota composition was not significantly different between the different diet groups (*p* = 0.24) (Additional file 3: Fig. S3), whereas, after prolongation of the diet intervention (3 weeks, 68 dpf) a significant association between the diet and the gut microbiota was detected (*p* = 0.018). The top-15 most discriminant genera associated with the diet induced microbiota difference were further investigated (Fig. 4B), revealing that these genera were absent in all fish after one week on the distinctive diets (Additional file 4: Fig. S4 and Additional file 5: Fig. S4 and Additional file 5: Fig. S4_2). This finding implies that short term diet exposure (1 week) is insufficient to elicit the diet induced microbiota changes. The relative abundances of the most discriminating genera of the gut samples were consistently zero or extreme low except for *Rhodobacter* and *Pseudomonas* (Additional file 4: Fig. S4), indicating that microbiota fluctuations in the zebrafish gut were not influenced to a larger extend by the surrounding water microbiota composition. RDA of the genera composition at 68 dpf associated *ZOR006* and unclassified Desulfovibrionaceae with fish fed a control diet, whereas associated *Mycobacterium*, *Vibrio*, *Aeromonas* and *Methylobacterium* with fish fed a saponin-supplemented diet and associated *Flavobacterium*, unclassified Sutterellaceae, *Bacteroides*, *Pandoraea*, *Rhodobacter*, unclassified Barnesiellaceae and *Plesiomonas* with fish fed butyrate-supplemented diet. The relative abundances of the most discriminative genera detected by the RDA (Fig. 4B, subset of boxplots around the RDA) together with the heatmap of the relative abundances of most important taxa (Additional file 6: Fig. S5) demonstrated distinct microbial profiles associated with butyrate and saponin-supplemented diets.

### Butyrate reduced taxa connectivity in the zebrafish gut

**Fig. 5:**
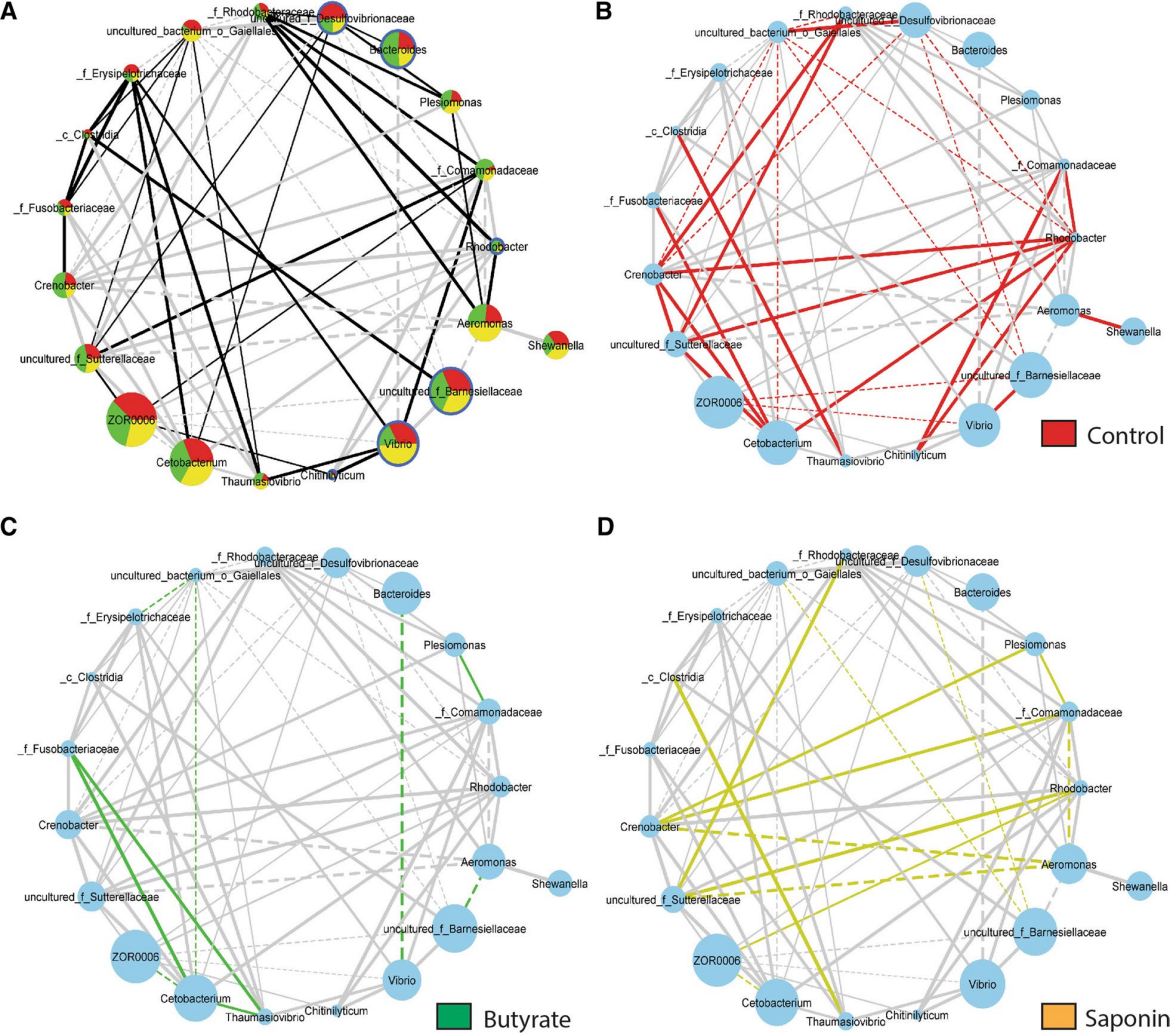
Taxa connectivity: taxa included when prevalence is ≥ in 3/10 samples, abundance is ≥ 10 counts in 1 M and significance ≤ 0.1. The lines inform about the nature of the taxa interaction: the thickness of the lines represents the strength of the correlation (r-score value) and the shape of the lines represents the direction of the correlation, straight lines mean positive correlation (co-occurrence) whereas dashed lines mean negative correlation of the pairs of taxa (anti-occurrence). **A** Pairs of taxa co- and anti-occurring for all the diets: in black the interactions occurring in all three diets whereas in grey the interactions not occurring in all diets. Node size corresponds to average abundance of taxa for all diets at 68 dpf. **B** In red the interactions occurring in the control fed fish and not in the other two diets. Node size corresponds to average abundance of taxa for control diet at 68 dpf. **C** In green the interactions occurring in the butyrate fed fish and not in the other two diets. Node size corresponds to average abundance of taxa for butyrate diet at 68 dpf. **D** In yellow the interactions occurring in the saponin fed fish and not in the other two diets. Node size corresponds to average abundance of taxa for saponin diet at 68 dpf

After assessing distinct microbiota composition due to diets after 3 weeks of feeding (68 dpf), taxa connectivity was analyzed by network analyses of co- and anti-occurrence of each pair of taxa at 68 dpf, based on the relative abundances (Fig. 5). The gut microbiota in fish fed the control diet presented a higher degree of taxa connectivity when compared to the gut microbiota of fish that were fed either the butyrate- or saponin-supplemented diet. Quantification of pairs of taxa with significant connectivity were compared using the cumulative frequency histogram (*p* < 0.05; *p*.log < 1.30, Additional file 7: Fig. S6A), showing an increase of connecting pairs of taxa in the control fed fish compared to saponin and butyrate fed fish at 68 dpf. These differences in taxa connectivity were not present after 1 week of feeding (54 dpf) and occurred exclusively after 3 weeks of feeding (68 dpf) where only control fed fish increased taxa connectivity from 54 to 68 dpf and not saponin and butyrate fed fish (Additional file  7: Fig. S6B).

### Gut transcriptome analysis reveals unique and shared effects of butyrate and saponin

**Fig. 6:**
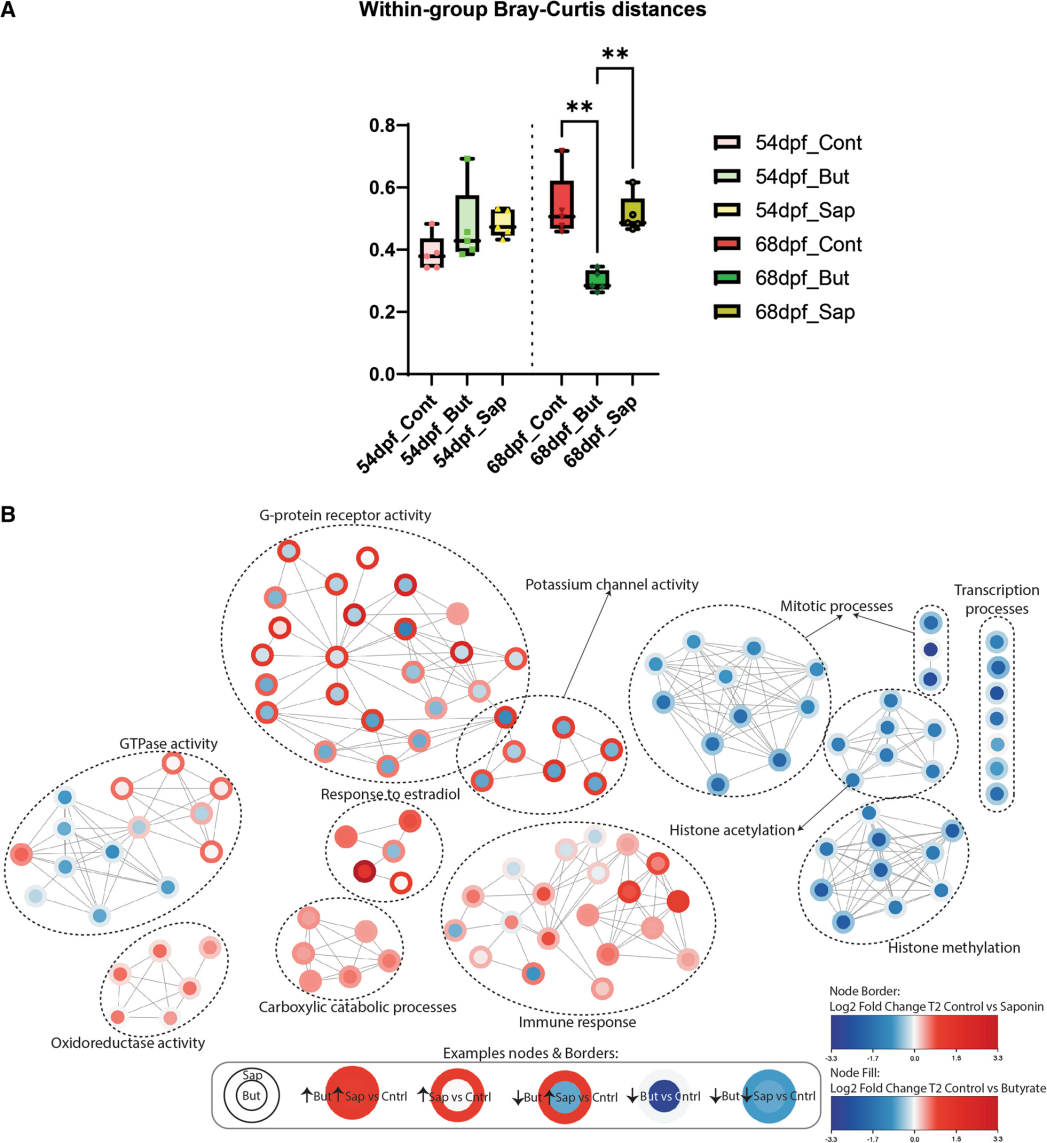

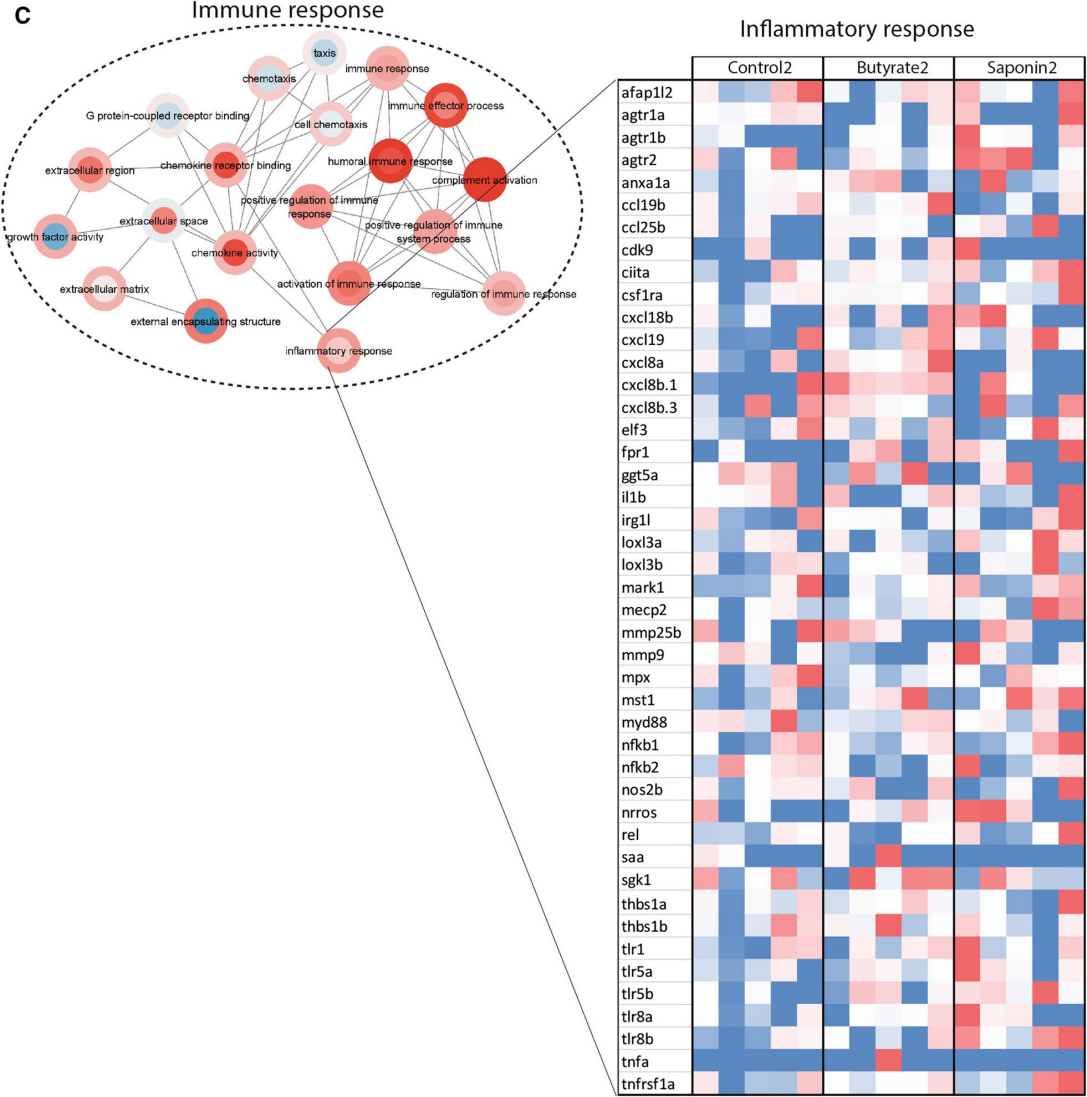
Effects of butyrate and saponin on the host gut transcriptome. **A** Bray–Curtis distances to examine the dissimilarity of the host transcriptome across diets and timepoints. ** *p* ≤ 0.01, Kruskal–Wallis test after testing for non-normally distributed data by Shapiro–Wilk test. Whiskers: min. to max. shall all points with median. **B** Network depicting transcriptomic regulation of butyrate and saponin supplemented diets vs control diet at 68 dpf. Each node is a GO term and the node border represent the log2 fold-change of the control diet vs the saponin supplemented diet and the node fill represent the log2 fold-change of the control diet vs the butyrate supplemented diet. The edges connect nodes containing at least 10 genes and sharing 50% of the contained genes. Related GO terms are encircled encompassing canonical pathways. Shared effects on the gut transcriptome can be observed when edge and fill of a node have the same color in the network: up-regulation -in red- and down-regulation -in blue- compared to the control feed. **C** Immune response-associated GO terms and particularly inflammatory response analysed in fish fed a control, butyrate and saponin diet. Genes are expressed in tpm and scaled colored per individual gene value. The heatmap contained genes color-scaled per individual gene that reflect the individual within group fish-to-fish variation

After observing substantial dietary induced differences in bacterial composition and taxa connectivity, transcriptome profiles of the same zebrafish gut samples were analyzed. This analysis (pipeline described in Fig. 2) resulted in a total of 47,046 genes expressed in transcripts per million (tpm). Within-group transcriptome differences across diets and timepoints revealed a significant difference of dissimilarity of the transcriptomic samples after 3 weeks of feeding (68 dpf) and not after 1 week of feeding (54 dpf). At 68 dpf fish fed the butyrate diet presented more significantly homogeneous gut transcriptomic profile than fish fed the control and the saponin diets (Fig. 6A).

Since the transcriptomic profiles were most dissimilar after 3 weeks of feeding the unique and shared effects of butyrate and saponin on the host gut were examined by creating a transcriptome network analysis (Fig. 6B**,** all raw data in Additional file 9). Compared to control fed fish, butyrate and saponin significantly down-regulated 893 GO terms while significantly up-regulated 40 GO terms out of a total of 6111 GO terms (Additional file 9). The transcriptomic network depicts a shared down-regulation of the transcription and mitotic processes as well as histone acetylation and histone methylation, that is most prominently observed in butyrate fed fish (Fig. 6B). Compared to control fed fish, both butyrate and saponin up-regulated the carboxylic catabolic processes, the oxidoreductase activity, response to estradiol and the immune response although some specific GO terms within these processes present differential modulation (Fig. 6B).

Compared to control fed fish, saponin up-regulated 37 GO terms that were down-regulated in butyrate fed fish while butyrate up-regulated 79 GO terms that were down-regulated in saponin fed fish (Additional file 9). Saponin up-regulated genes associated to GTPase activity, potassium channel activity and G-protein receptor activity which were down-regulated in butyrate fed fish (Fig. 6B). G-protein receptor activity is the GO term category that shows the strongest opposite regulation between saponin (up-regulated) and butyrate (down-regulated) and encompassed GO terms associated to photoreceptor activity, serotonin receptors activity as well as synaptic signaling (Fig. 6B). To explore the immune-related effects of butyrate and saponin, the immune response of the transcriptome network was zoomed in on (Fig. 6C). In particular, the GO term “inflammatory response” was examined for butyrate and saponin fed fish. Compared to controls, saponin and butyrate up-regulated genes involved in the “inflammatory response” associated to chemokine activity as well as leukocyte and innate cell recruitment (*ccl19b, ccl25b, csfr1ra, cxcl18b. cxcl19, cxcl8a, cxcl8b.1, cxcl8b.3, fpr1, mpx, mst1* and *tlr-*family) (Fig. 6C).

### Gut quantitative histological analysis depicted distinct gut architectural profiles for butyrate and saponin

**Fig. 7:**
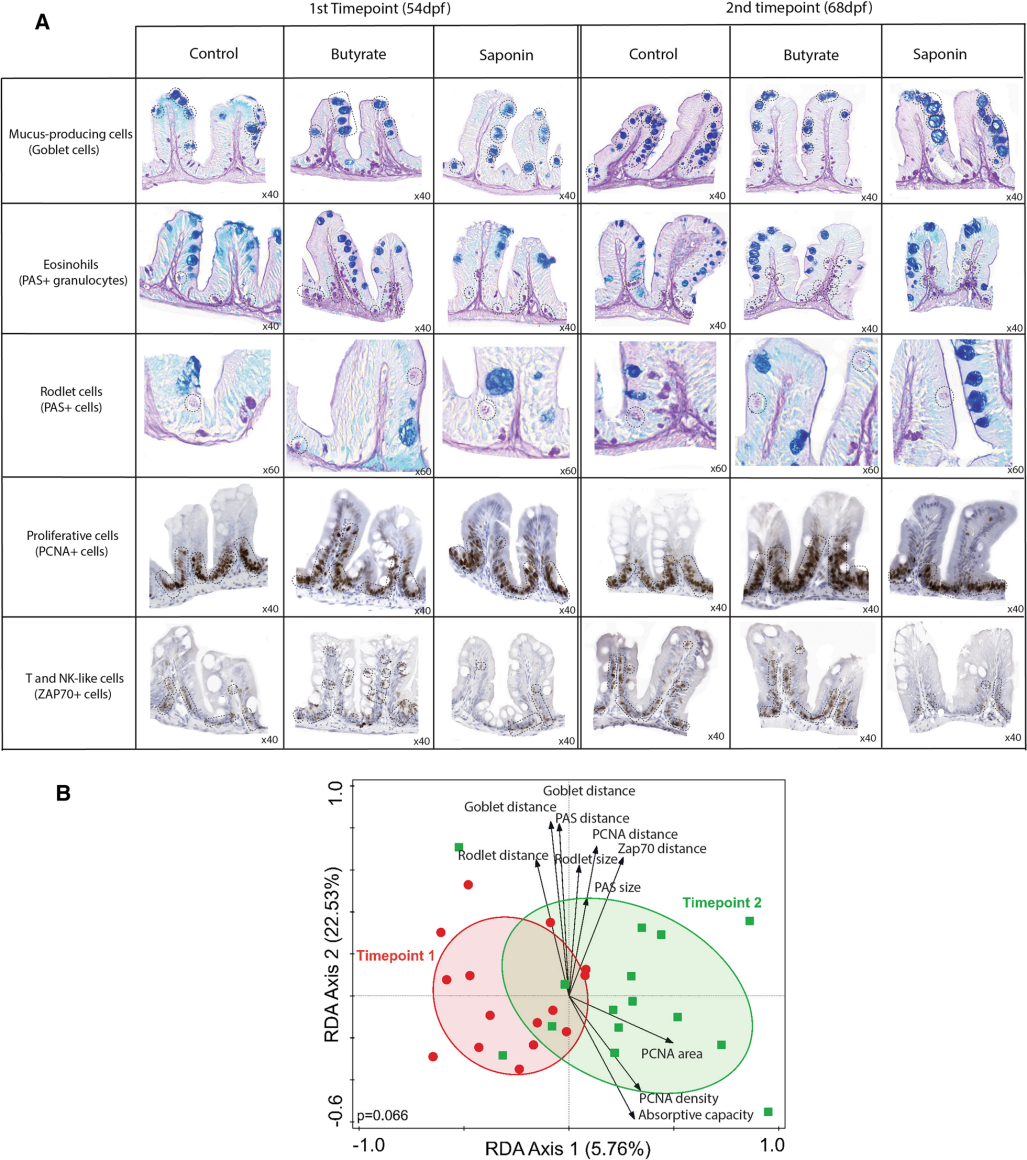

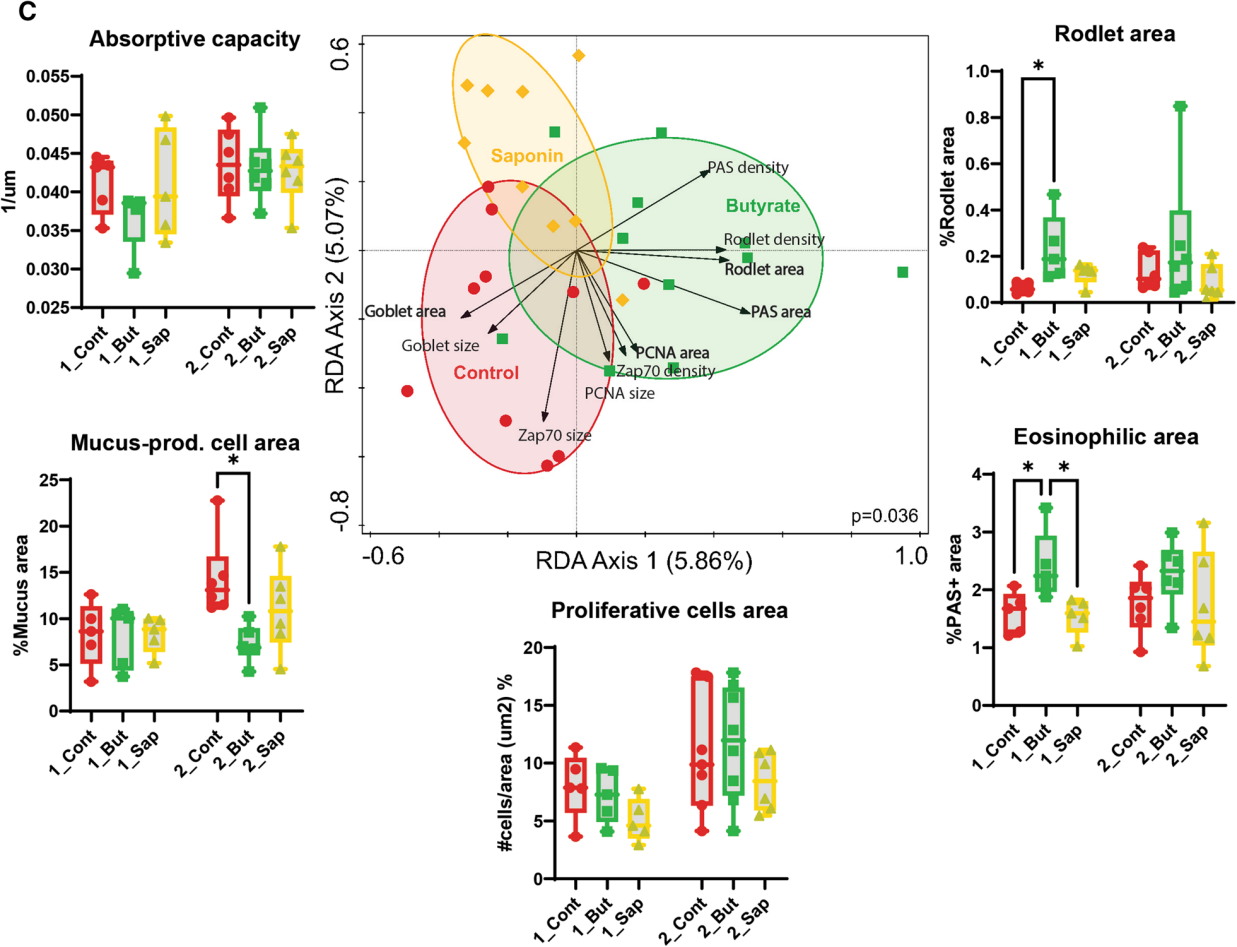
High-throughput quantitative histological analysis. **A** Representative pictures of all cell-types analyzed for all the diets and timepoints with cells of interest in dashed black lines per each group. **B** Redundancy analysis to examine the effect of time on the histological parameters analyzed. The x axis separated the samples by timepoints and explained 5.76% of the variation observed. The link of time and variation of the histological parameters was not significant (*p* = 0.066) **C** Redundancy analysis to examine the effect of diet on the histological parameters analyzed. The x axis separated the samples of butyrate fed fish from saponin and control fed fish and explained 5.86% of the variation explained. The y axis separated saponin fed fish from control fed fish and explained 5.07% of the variation observed. The link of time and variation of the histological parameters was significant (*p* = 0.036). The top 10 most distinctive histological parameters are depicted in black arrows. The direction of the arrows correlate with the dietary intervention and the length of the arrows represents the strength of the correlation. The boxplots around the RDA depicted the absorptive capacity and the percentage area of cells of interest compared to the total gut area per diet and timepoint. *p ≤ 0.05, one-way ANOVA test or Kruskal–Wallis test after testing for normality on data distribution by Shapiro–Wilk test. No false discovery rate performed. Whiskers: min. to max. shall all points with median

The zebrafish gut samples collected were analyzed using high-throughput quantitative histological analysis. While microbiota and transcriptomic data showed differential as well as similar effects of the butyrate and the saponin supplementation, the tissue make-up and topography provided further insight on whether changes in gene expression and microbiota also coincide with morphological indications of disturbed intestinal host gut health. Whole images were obtained from scanned slides and quantification was automated for several parameters: the AC and the cell area fraction, cell density, cell size, and the distance of each individual cell to the outer serosal layer for cell lineages of particular interest, including mucus cells (goblet cells), eosinophils (PAS + granulocytes), rodlet cells (PAS +), proliferative cells (PCNA + cells) and T and NK-like cells (Zap70 + cells). Representative pictures of all cell-types and time-points are shown in Fig. 7A.

The effect of time did not correlate to any of the histological parameters analyzed (black arrows in the RDA graph, Fig. 7B, *p* value = 0.066) except for the increase of the PCNA area over time, indicative that the relative number of proliferative cells increased during fish development. Significant differences on the histological gut parameters were found due to the dietary interventions (*p* = 0.036) (Fig. 7C). The absorptive capacity of the fish gut was decreased for the butyrate fed fish compared to saponin and control fed fish at 54 dpf, although displayed similar values at 68 dpf (boxplots around Fig. 7C). The area of the eosinophils and rodlet cells increased in butyrate fed fish compared to saponin and control fed fish after 1 week of feeding (54 dpf), suggesting an inflammatory condition which was partly alleviated but not fully resolved at 68 dpf. In addition, compared to controls, fish fed the butyrate diet showed a clear mucus-producing cell depletion after 3 weeks of feeding (68 dpf). Saponin fed fish presented a reduced proliferative cell area compared to butyrate and control fed fish. To illustrate these differences in histological parameters, accepting the biological fish to fish variation within each group, a heatmap of each individual fish and all the histological parameters per each RDA axis was generated (Additional file 8: Fig. S7). The combination of these observations suggested an acute inflammatory response (after one week of exposure) of the fish fed the butyrate diet by increased eosinophils, rodlet cells and a decrease of the AC. The inflammatory condition remain unresolved after 3 weeks of feeding (68 dpf) as the fish fed the butyrate diet still presented increased eosinophils and rodlet cells and a depletion of mucus cells compared to saponin and control fed fish (black arrows and boxplots Fig. 7C, representative pictures Fig. 7A).

### Combinatorial approach reveals distinct profiles for saponin and butyrate fed fish

**Fig. 8:**
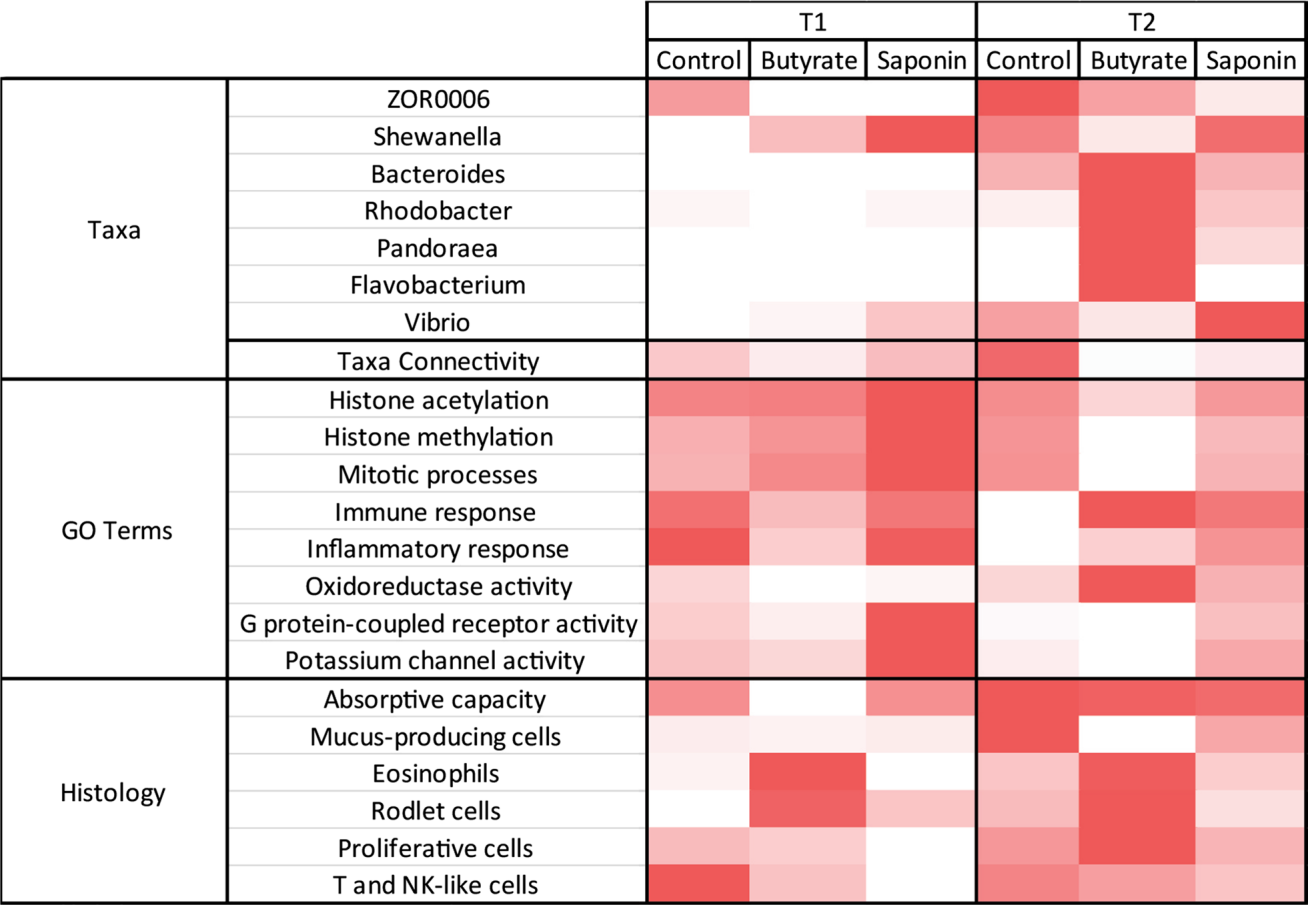
The heatmap brings together the main observations of each analysis and compare them per diet and timepoint. The more representative genera are illustrated with the average relative abundance per timepoint and diet. The taxa connectivity contained the amount of pairs of taxa that correlate to each other in a significant fashion ( *p* ≤ 0.05). The GO terms contain the transcripts per million (tpm) of all genes expressed in the dataset that collapsed under that GO term. All histological parameters are normalized and scaled (from 0 to 1). Each individual feature within the heatmap is normalized and colored from red (more present) to white (absent)

In order to define robust and multi-parameter supported effects of butyrate and saponin supplementation, the key findings of the different datasets were integrated in a heatmap (Fig. 8). Control fed fish did not present extreme microbiota fluctuations over time. Butyrate fed fish presented the most divergent microbiota composition (with increased relative abundance of *Bacteroides, Rhodobacter, Pandoraea* and *Flavobacterium*) and the lowest taxa connectivity compared to the other diets, which might be indicative of disturbed ecosystem stability. Saponin fed fish presented an increased number of *Vibrio* contrasting with butyrate fed fish. Compared to control fish, butyrate and saponin shared an increased expression of genes associated to immune responses, inflammatory responses and oxidoreductase activity. Besides, butyrate fed fish presented down-regulated genes in GO terms associated with histone acetylation, histone methylation, mitotic processes and G-protein coupled receptor activity. These differential gene expressions patterns were stronger after 3 weeks of feeding (68 dpf) compared to 1 week of feeding (54 dpf). In terms of histology, after 1 week of feeding butyrate, fish already showed increased area of eosinophils and rodlet cells compared to saponin or control fed fish, which is consistent after 3 weeks of feeding. Butyrate fed fish at 68 dpf showed decreased area of mucus cells compared to control fed fish. The histological parameters for saponin fed fish appeared to be less pronounced than those of butyrate fed fish. Collectively, these observations, showed fish fed a butyrate supplemented diet elicited a stronger response in terms of changes in the microbial composition, expression of genes associated to immune activation processes as well as the presence of (pro)inflammatory-like cells such as eosinophils and rodlet cells and depletion of mucus cells.

### Butyrate and saponin increased neutrophil and macrophage recruitment in the gut of zebrafish larvae

**Fig. 9:**
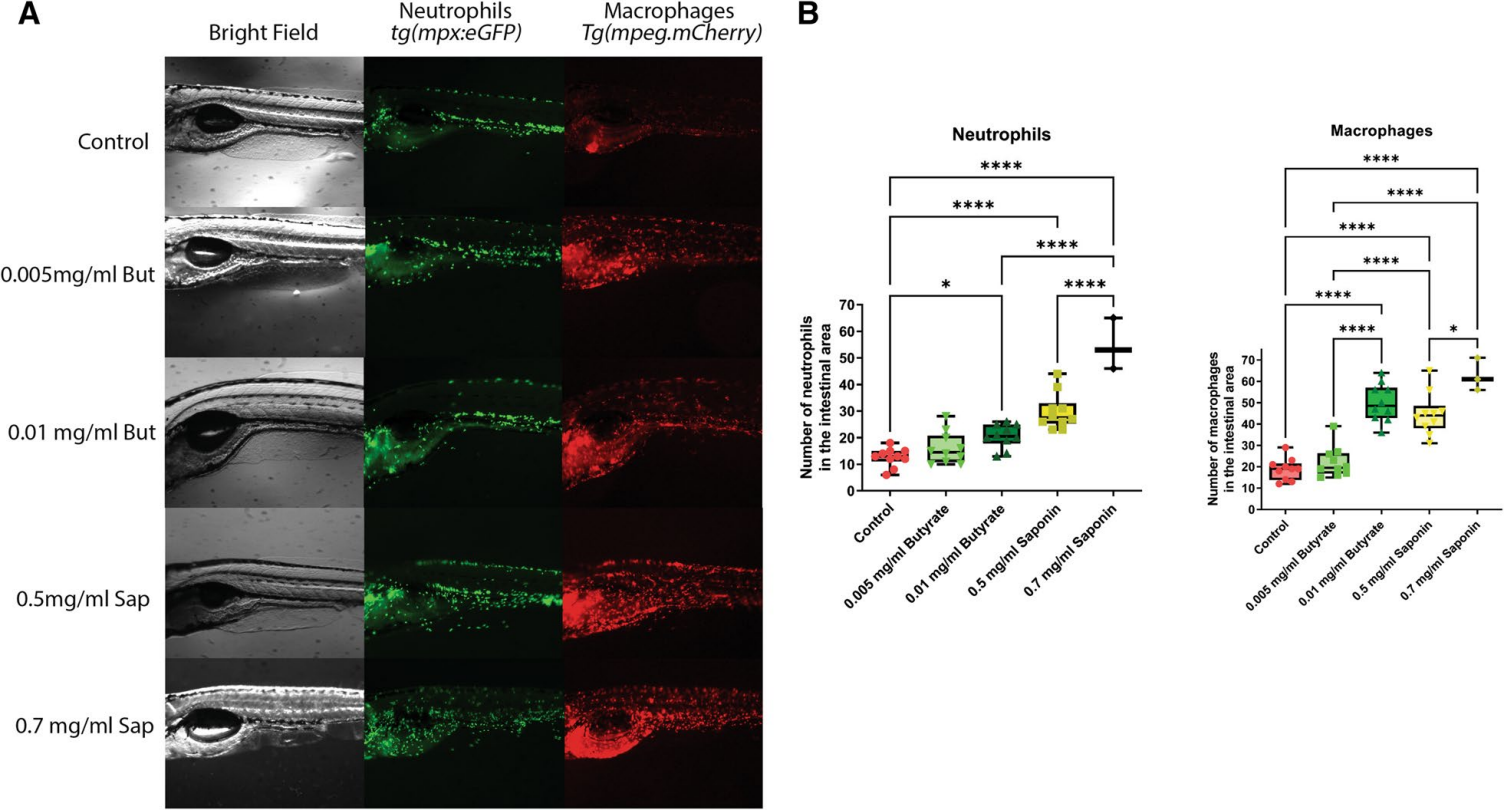
**A** Representative pictures of the fluorescent in vivo imaging of the gut area of Tg(mpeg1:mCherry / mpx:eGFPi^114^) larvae exposed to control media, 0.005 mg/ml and 0.01 mg/ml butyrate and 0.5 mg/ml and 0.7 mg/ml saponin. **B** Quantification of neutrophils and macrophages in the gut area of the zebrafish larvae (n = 10 in all groups except 0.7 mg/ml saponin where n = 3). *p ≤ 0.5, **** *p* ≤ 0.0001 Kruskal–Wallis test after testing for non-normally distributed data by Shapiro–Wilk test. Whiskers: min. to max. shall all points with median

Since the data clearly indicated an unexpected induction of immunity related functions upon butyrate addition to the feed (Fig. 6B), the advantages of the zebrafish model system were used to validate the results by in vivo imaging of fluorescently labeled neutrophils and macrophages upon butyrate and saponin exposure in zebrafish larvae. Double Tg(mpeg1:mCherry/mpx:eGFPi^114^) zebrafish larvae were exposed to butyrate and saponin in different doses for 3 days (3-6dpf) and were (in vivo) imaged at 6dpf. Fish treated with butyrate as well as saponin presented a dose-dependent increase of neutrophils and macrophages in the intestinal area (Fig. 9A). The quantification of the cells present in the gut area showed that butyrate as well as saponin significantly increased neutrophils and macrophages in the gut of zebrafish larvae (Fig. 9B).

## Discussion

In the present study the effects of butyrate and saponin-supplemented feed in the zebrafish gut were assessed following a combinatorial approach that integrates several datasets and validating the results by in vivo imaging. Juvenile zebrafish fed a butyrate-supplemented feed for 3 weeks presented a modulated microbial composition and low taxa connectivity, increased expression of genes associated with immune response together with an increased eosinophil and rodlet cell presence and mucus-producing cell depletion in the gut tissue. Moreover, butyrate increased the neutrophil and macrophage in vivo recruitment to the gut area in transgenic zebrafish larvae. Zebrafish fed a saponin-supplemented diet showed differentially modulated microbial composition from butyrate and low taxa connectivity as well as increased expression of immune response while the histological parameters comparable to control fed fish. The combinatorial approach of bacterial microbiome profiling, host gut transcriptomics, automated high-throughput quantitative histology (novel in zebrafish research) together with in vivo innate cell recruitment in the gut area in zebrafish larvae revealed evidence of the pro-inflammatory effects exerted by butyrate supplementation which were partly shared with the well-establish pro-inflammatory saponin supplementation, indicating detrimental effects of butyrate in the zebrafish intestinal milieu.

While saponin and soybean meal have been consistently associated with gut inflammation in several fish species, including carp [80, 81, 96], salmon [5, 32, 37, 40, 80, 81] and zebrafish [30, 49], butyrate has been reported to convey beneficial effects when supplemented to fish feed such as intestinal growth enhancement [71] and immunostimulant and antioxidant properties [20, 47], (reviewed in [1]. However, previously published studies with histological and gene expression redouts compared butyrate supplementation to other challenges such as high concentration of plant-based meal or a pathogen challenge. As a matter of fact, butyrate-supplementation effects depended on co-treatment(s) employed and duration of the feeding intervention. For instance, 0.8% inclusion of sodium butyrate in a low percentage plant-containing diet in gilthead sea bream for 10 weeks resulted in a mild inflammatory reaction whereas in the same study, 0.4% inclusion of sodium butyrate in high percentage plant-based diet for a longer period protected the host during a bacterial challenge [65]. No baseline studies of butyrate-supplemented feed are reported in the (zebra)fish literature to our knowledge. In the present study, we used for the first time a butyrate-supplemented diet for zebrafish and our combinatorial approach showed that 0.01% inclusion of butyrate induced a pleiotropic damaging response comparable to the well-establish pro-inflammatory anti-nutritional factor soy saponin. In mammals, colonocytes located along the gut crypts take up the butyrate produced by the microbiota, preventing high concentrations of this SCFA to reach the proliferating stem cells at the bottom of the crypts. In fact, high concentrations (1.5–2 mM) of butyrate were shown to be toxic to mouse pluripotent stem cells in vitro [45]. This is especially relevant in cryptless organisms such as fish [3, 41, 82], where higher concentrations of butyrate can reach the stem cells localized between the intestinal folds (villi). Mechanistic studies in mouse and zebrafish larvae suggested that butyrate at high concentrations inhibits stem cell proliferation via FoxO3 in cryptless organisms such as zebrafish [25]. Taking these observations together, the butyrate-supplemented diet showed a compromised intestinal epithelial barrier function, coinciding with a disrupted microbiota composition with decreased taxa connectivity, and increased expression levels of genes associated with inflammatory and immune responses. The inflammatory response due to butyrate was confirmed by enhanced innate immune cell recruitment *in* vivo to the zebrafish gut. Our data warrants that further research should investigate the long term effects of butyrate-supplemented feed and susceptibility towards infectious or inflammatory challenges which were not investigated here. Potentially, butyrate-associated immuno-stimulation early in life, could boost immunity and strengthen disease resistance in later life stages (trained immunity) [64].

Disruption of the gut microbiota homeostasis, often caused by an imbalance in the microbial community (or dysbiosis), is commonly associated with inflammatory conditions in the zebrafish gut [8, 11] also (reviewed in [12, 50]). However, whether these disruptions of microbial community cause gut inflammation in a direct manner or via disruptions in the microbiota is a matter of discussion and current research in the fish immunity and nutrition field. Next to investigating the community composition, more information might be extracted from the analysis of microbial networks. For example, in inflammatory bowel disease patients, topological properties of the co-occurring bacterial networks identified anti- and pro-inflammatory key organisms that defined the degree of structure of the ecosystem [6]. In fish, recent studies validated the usage of co-occurrence and anti-occurrence taxa networks to identify the core gut European seabass microbiota [38] as well as to reveal microbial interactions due to prebiotics and probiotics [53]. In the present study, zebrafish fed 3 weeks a butyrate-supplanted feed presented altered the microbiota composition as well as reduced taxa connectivity (co- and anti-occurrence) compared to control (and to a lesser extent to saponin)-fed fish (Figs. 4, 5 and Additional file 7: Fig. S6). We had 6 tanks, 2 per each diet and 1 tank per diet was sampled after 1 week of feeding intervention and the other after 3 week of feeding intervention. This could have an effect on the sample clustering of the fish microbiota. However, the water from the recirculating system was the same for all tanks and its quality remained comparable in all tanks across the study (Additional file 2: Fig. S2). Furthermore microbiota composition of the water samples of the fish tanks were comparable among themselves and very different from the gut samples (Additional file 4: Fig. S4 and Additional file 5: Fig. S4_2) suggesting that the differences in microbial communities found across dietary treatments did not originate due to the separate environments per treatment. Butyrate increased the relative abundance of the genera *Rhodobacter, Flavobacterium* and *Bacteroides* that were previously associated with gut inflammation in fish [51, 78, 94] whereas saponin increased the relative abundance of the *Vibrio* genus, which contains several pathobiont species which might become pathogenic upon challenge of the gut barrier integrity (reviewed in [15]. In mammals, butyrate is produced by fermenting bacteria in the intestinal tract and until now scientists were not able to measure any naturally occurring concentrations of butyrate in the zebrafish gut [14]. Since it is not certain whether fish gut may produce butyrate, exogeneous butyrate supplementation may disrupt the growth of bacteria since they may not be used to metabolize such substrate. In butyrate-fed fish increased abundance of *Bacteroides* correlated with lower abundance of *Vibrio*. Interestingly, in vitro studies have revealed that butyrate exposure can negatively impact the colonization of specific *Vibrio* c*ampbellii* PUGSK8 by its effect on biofilm formation capacity in these bacteria [36]. Taken together, these findings warrant further studies to understand the mechanisms by which butyrate influences microbial ecosystems.

Inflammatory-associated taxa in butyrate-fed fish matched with an increased expression of genes belonging to inflammatory and immune responses (Fig. 8). While targeted gene expression is commonly used in (fish) nutrition studies, this approach is often hypothesis-driven and the discovery risk of novel premises is relatively low compared to more comprehensive transcriptome analyses. In the present study, butyrate down-regulated genes associated with mitotic and transcription processes which is in line with the inhibition of stem cell proliferation previously reported [25], although proliferative cells (PCNA +) were not decreased in butyrate-fed fish as shown by the histological dataset. Butyrate down-regulated genes associated with histone modifications (acetylation and methylation) in line with previously described epigenetic effects of butyrate in mammals (reviewed in [31]. Further research may elucidate whether there is an effect of butyrate supplemented feed on epigenetic markers and in the affirmative case whether such epigenetic modifications can be passed on the fish offspring. A clear subset of chemokines within the inflammatory response appeared to be up-regulated after butyrate-supplemented feeding (Fig. 6C) among which *cxcl8a, cxcl8b.1* and *cxcl8b.3*. *Cxcl8* (or *il8*) is known as one of the most potent chemoattractant molecules for recruiting neutrophils (expressing CXCR1/2 receptors for *Cxcl8*) and other leukocytes upon inflammation [60]. Although IL8 did not affect human eosinophils in vitro [63], eosinophils are able (via granule proteins) to stimulate neutrophils that produce IL8 and superoxide contributing to gastrointestinal pathologies [72]. However, eosinophil research in the context of gastrointestinal health is limited in humans and mice [34] as well as (zebra)fish [7]. Butyrate increased eosinophil and rodlet cell area even after 1 week of feeding, while reduced the presence of mucus cells overtime (Fig. 7), features associated with (chemically-induced) intestinal inflammation in zebrafish [11]. Rodlet cells were first reported to act against fish parasites and later studies disclosed their granulocyte nature and include them as part of the innate fish immune system, increasing in number when exogeneous stressors were present [18, 33, 54, 69]. More research into this well-known but often forgotten cell type may elucidate its role in (zebra)fish mucosal immunology.

To reinforce the observation that saponin and butyrate recruited immune cells to the gut we used transgenic zebrafish larvae to in vivo visualize neutrophil and macrophage presence in the gut. The fact that saponin induced a stronger cell recruitment than butyrate could be explained by the fact that lower concentrations of butyrate were used (mimicking the ones employed in the diets) (Fig. 9). Other studies showed decreased neutrophil recruitment after tail wounding when zebrafish larvae were immersed to butyrate [14]. However, such studies briefly immersed zebrafish larvae to extremely high concentrations of sodium butyrate (30 mM = 3303 mg/ml) and such study design may greatly differ from the naturally occurring physiological situation in the zebrafish gut. We hypothesize that the increased chemokine expression in butyrate fed fish might be the driving force for the increased leucocyte recruitment in the gut and further research may disclose specific butyrate modes of action in the (zebra)fish gut.

In the present study butyrate-supplemented feed appeared to modulate the microbial composition as indicated by low taxa connectivity, increased expression of gene associated to inflammatory processes as well as increased presence of rodlet cells, and eosinophils while decreasing Goblet cells. Moreover, we supplemented this data with in vivo observations of the increased recruitment of the neutrophil and macrophage population in the gut upon butyrate and saponin exposure. The combination of these datasets indicate that butyrate has fish-specific effects on the gut homeostasis that differ from the mammalian counterparts [28]. The particular fish gut structure, lacking intestinal crypts could play an important role on the absorption and the effect of the butyrate on the epithelial lining where chemokines might orchestrate the inflammatory-like response. However, in the present study butyrate absorption by the enterocytes in the zebrafish gut has not been quantified and should be addressed in future research. While more mechanistic studies are needed to shed light on the specific modes of action of butyrate on the fish gut health, the present combined study (omics, histology and imaging) provides evidence to support non-beneficial effect of butyrate-supplemented feed on growing juvenile zebrafish.

In conclusion, combining several high throughput approaches we provide a more comprehensive and granular view of the effects of dietary interventions on fish gut health. Translation to aquaculture species is possible since our redouts do not depend on any species-specific antibodies. However, integration of multi-layered high-throughput studies remain a challenge in fish because of various reasons. On the one hand, there are difficulties to fully comprehend the connections between the complex layers of data deriving from high-throughput methods and the most relevant outcomes (fish health biomarkers). On the other hand, scientist may not have yet the technology to adequately obtain multi-omics data with sufficient resolution (lack of noise) and reproducibility that facilitates omics datasets combination. In the present study, the detrimental effects of butyrate towards the zebrafish gut were congruent throughout all the datasets in our combinatorial approach strengthening the biologically relevant observation that butyrate appears detrimental to the zebrafish gut. Steps towards observational scientific studies with an integrative view, combining high-throughput datasets with imaging techniques to understand complex multifactorial biological processes such as fish gut health may help researchers to evaluate novel diets for healthier fish generations.

## Supplementary Information

**Additional file 1: Fig. S1.** Standard length (mm) was measured at 40, 54 and 68 dpf for the 3 diets by using a digital calliper.

**Additional file 2: Fig. S2.** Water quality values just before and during the experiment at 38, 45, 50, 56, 62 and 65 dpf. In green the range of preferable values for the measurements and in red the values above which the water quality is considered to be detrimental for the fish according to manufacturer’s instructions: A) pH (accepted range 6.6-8.4), B) Water conductivity (accepted range 300-1500 µS/m), C) Nitrite (accepted range 0-7 mM), D) Ammonium (only 0 mM accepted), E) Nitrate (accepted range 0-70 mM), F) Chlorine (only 0 mM accepted), G) General hardness (accepted range 2-16) and H) Carbonate hardness (accepted range 1.5-10).

**Additional file 3: Fig. S3.** Redundancy Analysis (RDA) at the 1st timepoint to examine the effect of the diets on the gut microbiota. The x axis separates saponin form control fed fish and explains 5.82% of the microbial differences observed and the y axis separates the butyrate form the saponin fed fish and explains 2.28% of the microbial differences observed. The top 15 most distinctive genera are depicted as supplementary variables in black arrows, p=0.24.

**Additional file 4: Fig. S4_1.** Relative abundances of the top 15 most distinctive genera for all diets at both timepoints, including water samples from all fish tanks at both timepoints.

**Additional file 5: Fig. S4_2.** Figure S4_1 continued.

**Additional file 6: Fig. S5.** Heatmap of the relative abundance (relative to 1) of the most distinctive and important taxa for all diets at the 2nd timepoint. Importance was calculated as (sqrt( CorS1^2+CorS2^2)), i.e., the length of the arrows in Figure 4B.

**Additional file 7: Fig. S6.** Normalized cumulative frequency histogram depicting the amount of significant pairs of taxa correlations per each diet A) at 68 dpf, B) at 54 dpf; (dotted line represents logarithmic p value =1.30 and p =0.05).

**Additional file 8: Fig. S7.** Heatmaps of each individual gut fish sample (n=5 diet / timepoint) for both axis of the redundancy analysis. Despite of the fish to fish variation present dietary effects are visible for both timepoints. Values are normalized and scaled from 0-1.

**Additional file 9:** Gene expression of transcripts from zebrafish gut fed either a control, butyrate- or saponin-supplemented diets at 54 and 69 dpf.

## Data Availability

Raw data of the transcriptomic analyses can be found in Additional file 9. All raw data will be uploaded to an open access repository.
