# Supplementary figures and images for: Omics and imaging combinatorial approach reveals butyrate-induced inflammatory effects in the zebrafish gut
Source: Anim Microbiome. 2023 Mar 3;5:15. doi: 10.1186/s42523-023-00230-2 (PMC9985269; doi:10.1186/s42523-023-00230-2)

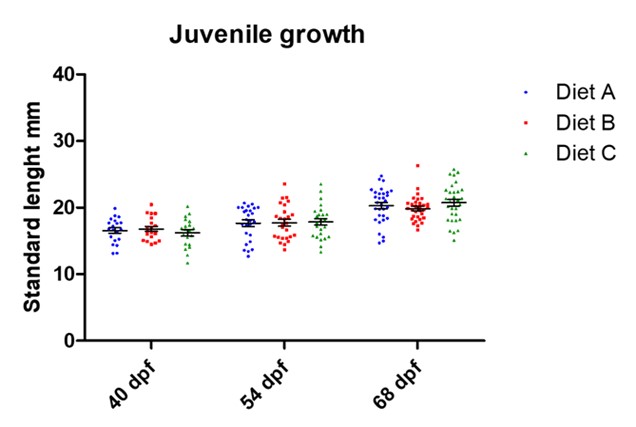

Supplement: Supplementary file 1 — Additional file 1: Fig. S1. Standard length (mm) was measured at 40, 54 and 68 dpf for the 3 diets by using a digital calliper. [file 42523_2023_230_MOESM1_ESM.jpg]

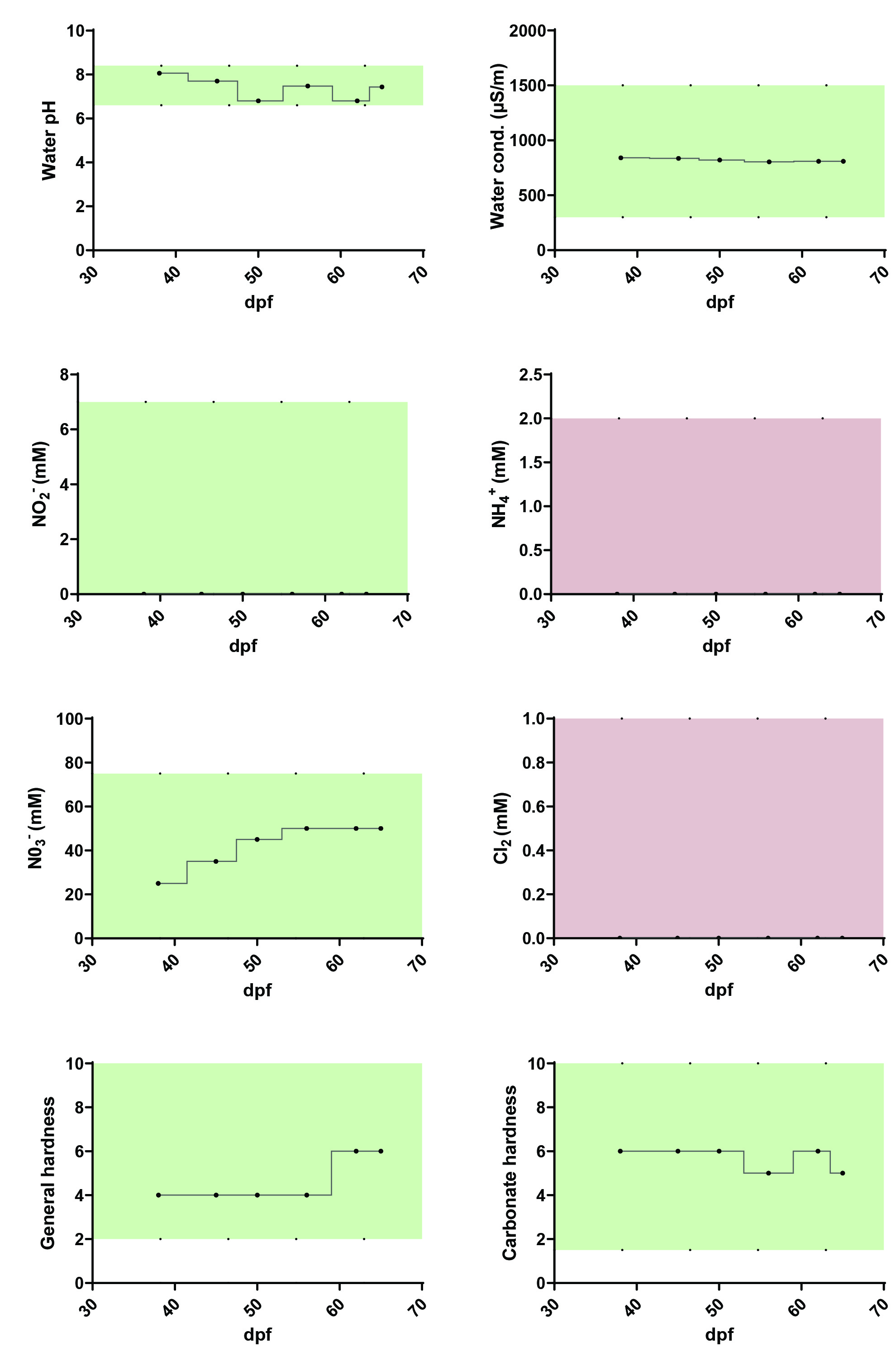

Supplement: Supplementary file 2 — Additional file 2: Fig. S2. Water quality values just before and during the experiment at 38, 45, 50, 56, 62 and 65 dpf. In green the range of preferable values for the measurements and in red the values above which the water quality is considered to be detrimental for the fish according to manufacturer’s instructions: A) pH (accepted range 6.6-8.4), B) Water conductivity (accepted range 300-1500 µS/m), C) Nitrite (accepted range 0-7 mM), D) Ammonium (only 0 mM accepted), E) Nitrate (accepted range 0-70 mM), F) Chlorine (only 0 mM accepted), G) General hardness (accepted range 2-16) and H) Carbonate hardness (accepted range 1.5-10). [file 42523_2023_230_MOESM2_ESM.jpg]

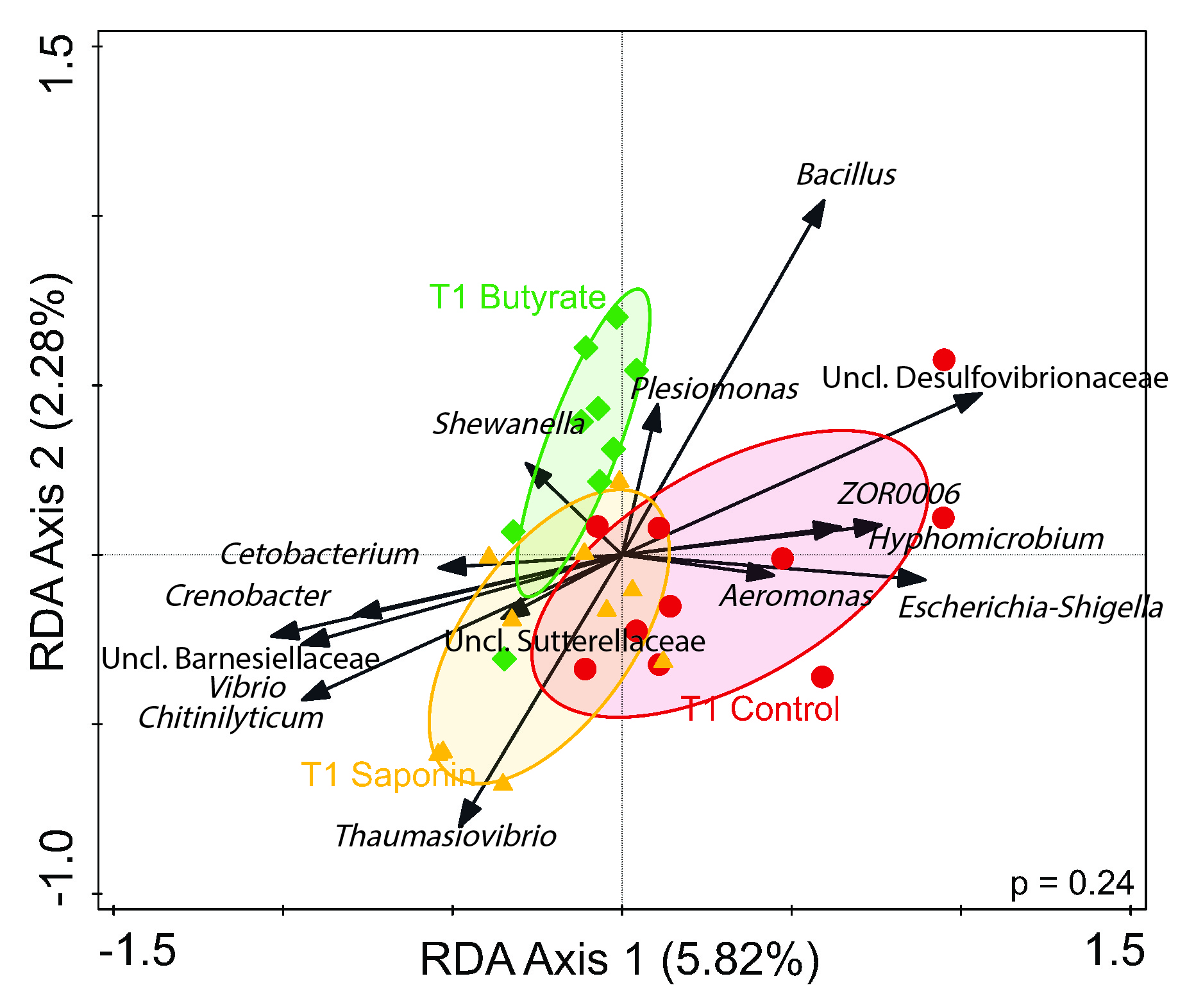

Supplement: Supplementary file 3 — Additional file 3: Fig. S3. Redundancy Analysis (RDA) at the 1st timepoint to examine the effect of the diets on the gut microbiota. The x axis separates saponin form control fed fish and explains 5.82% of the microbial differences observed and the y axis separates the butyrate form the saponin fed fish and explains 2.28% of the microbial differences observed. The top 15 most distinctive genera are depicted as supplementary variables in black arrows, p=0.24. [file 42523_2023_230_MOESM3_ESM.jpg]

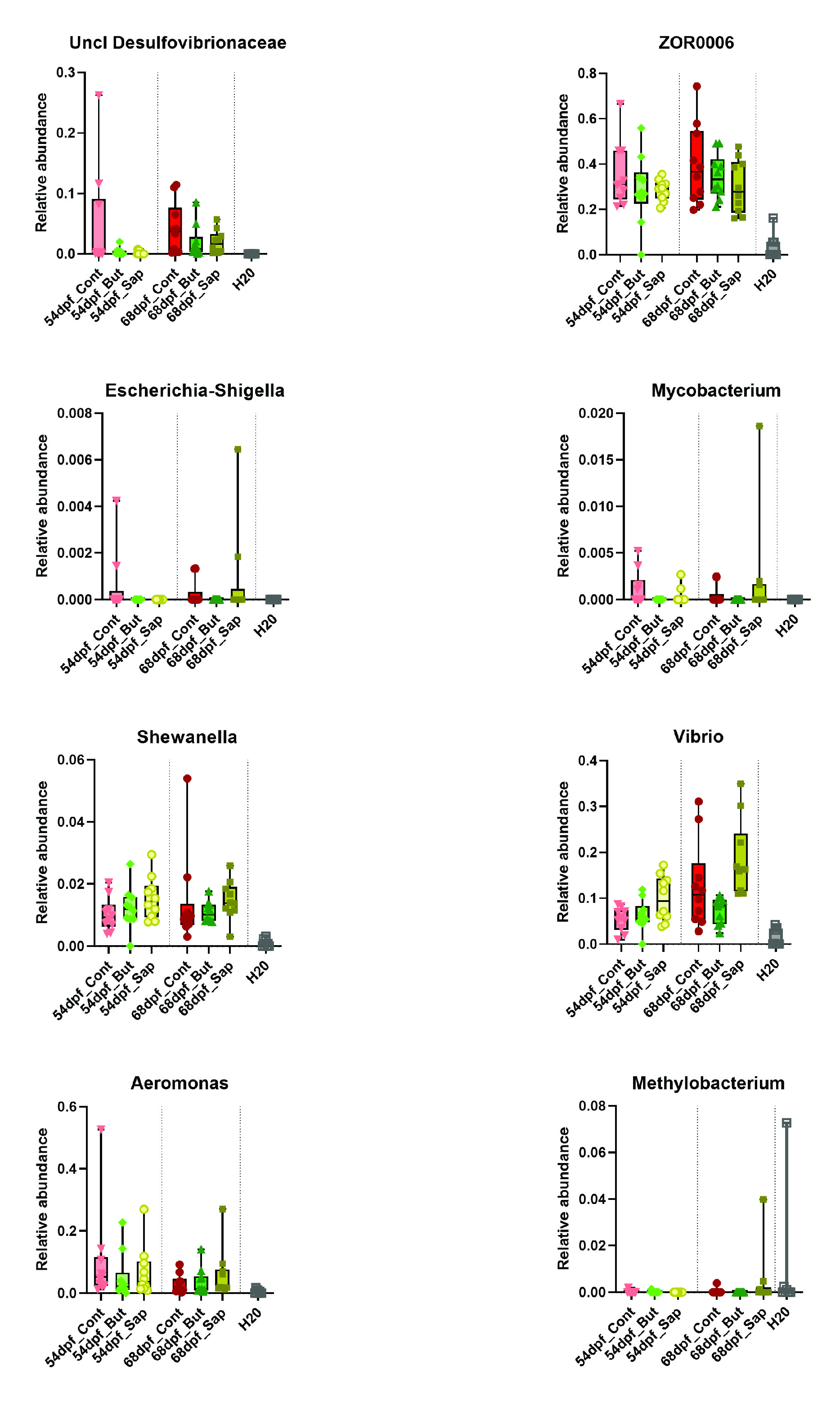

Supplement: Supplementary file 4 — Additional file 4: Fig. S4_1. Relative abundances of the top 15 most distinctive genera for all diets at both timepoints, including water samples from all fish tanks at both timepoints. [file 42523_2023_230_MOESM4_ESM.jpg]

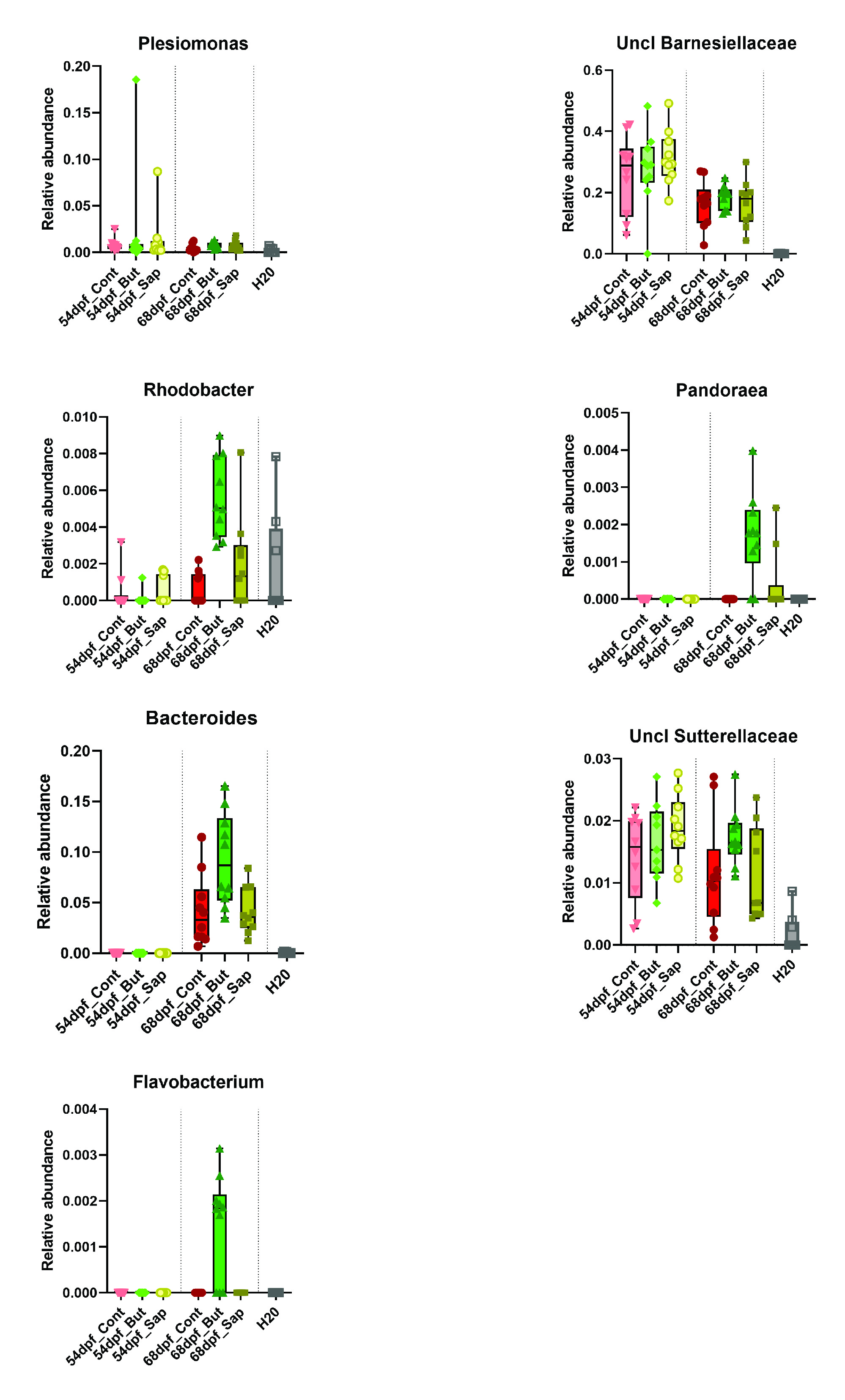

Supplement: Supplementary file 5 — Additional file 5: Fig. S4_2. Figure S4_1 continued. [file 42523_2023_230_MOESM5_ESM.jpg]

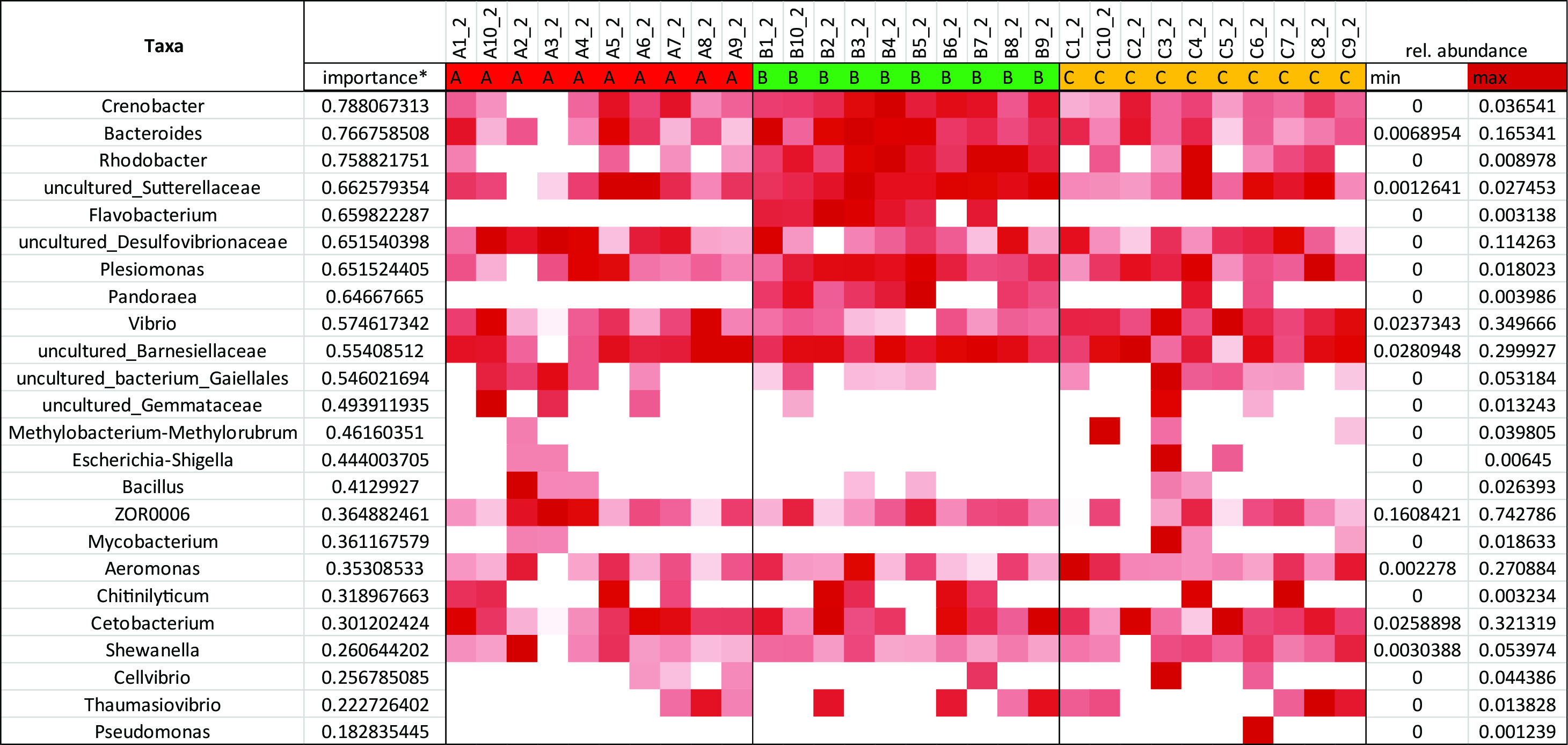

Supplement: Supplementary file 6 — Additional file 6: Fig. S5. Heatmap of the relative abundance (relative to 1) of the most distinctive and important taxa for all diets at the 2nd timepoint. Importance was calculated as (sqrt( CorS1^2+CorS2^2)), i.e., the length of the arrows in Figure 4B. [file 42523_2023_230_MOESM6_ESM.jpg]

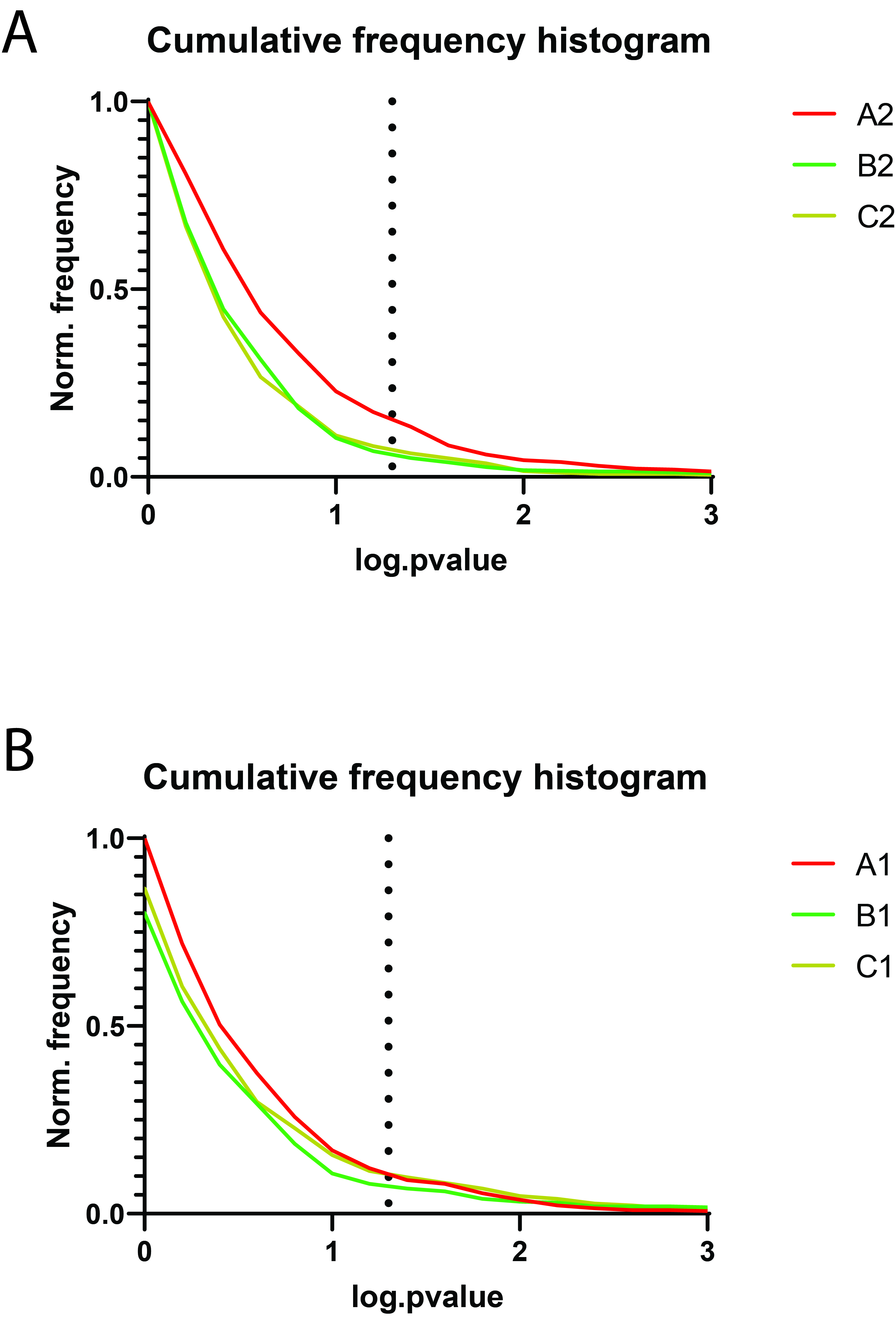

Supplement: Supplementary file 7 — Additional file 7: Fig. S6. Normalized cumulative frequency histogram depicting the amount of significant pairs of taxa correlations per each diet A) at 68 dpf, B) at 54 dpf; (dotted line represents logarithmic p value =1.30 and p =0.05). [file 42523_2023_230_MOESM7_ESM.jpg]

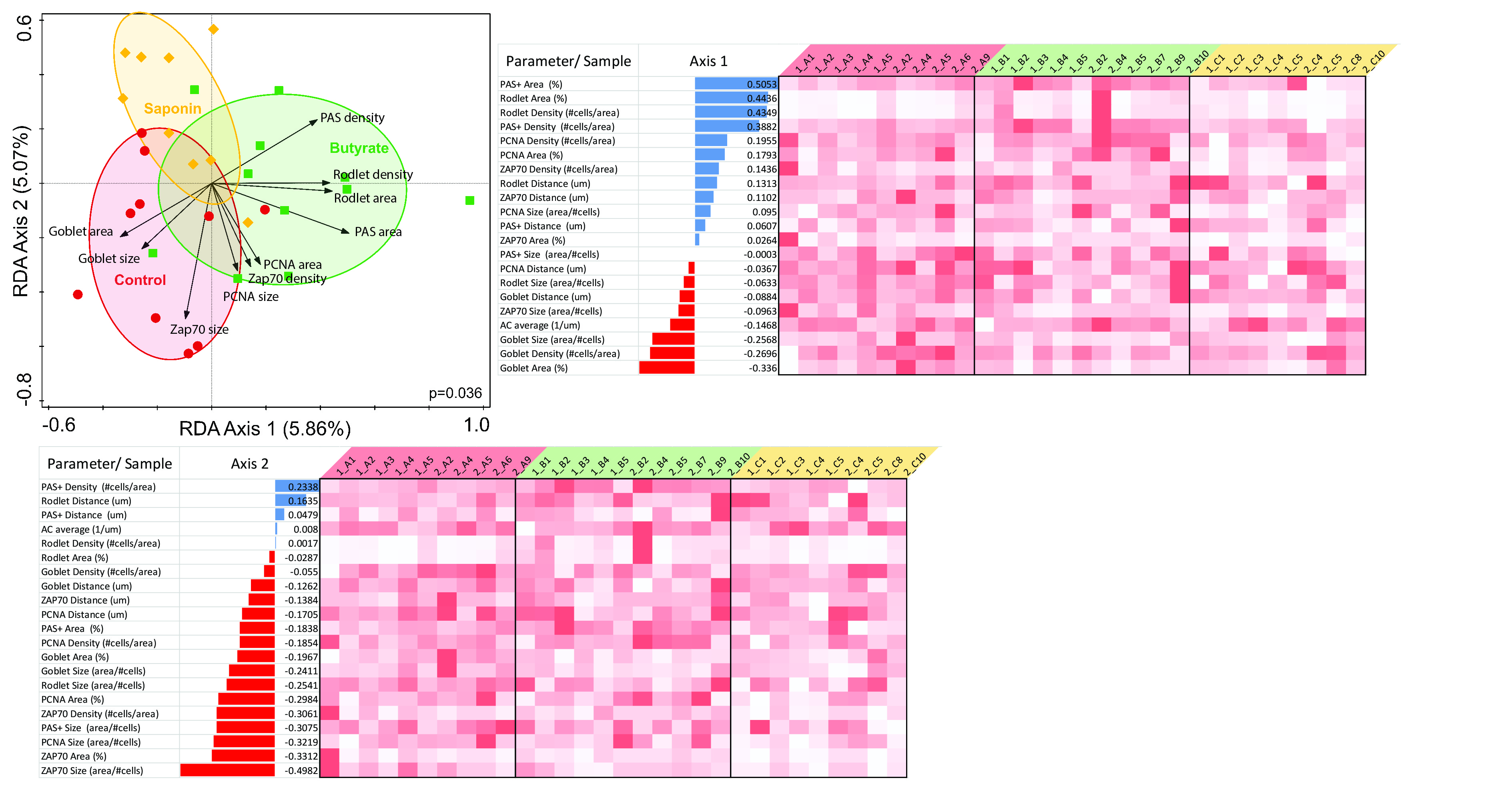

Supplement: Supplementary file 8 — Additional file 8: Fig. S7. Heatmaps of each individual gut fish sample (n=5 diet / timepoint) for both axis of the redundancy analysis. Despite of the fish to fish variation present dietary effects are visible for both timepoints. Values are normalized and scaled from 0-1. [file 42523_2023_230_MOESM8_ESM.jpg]

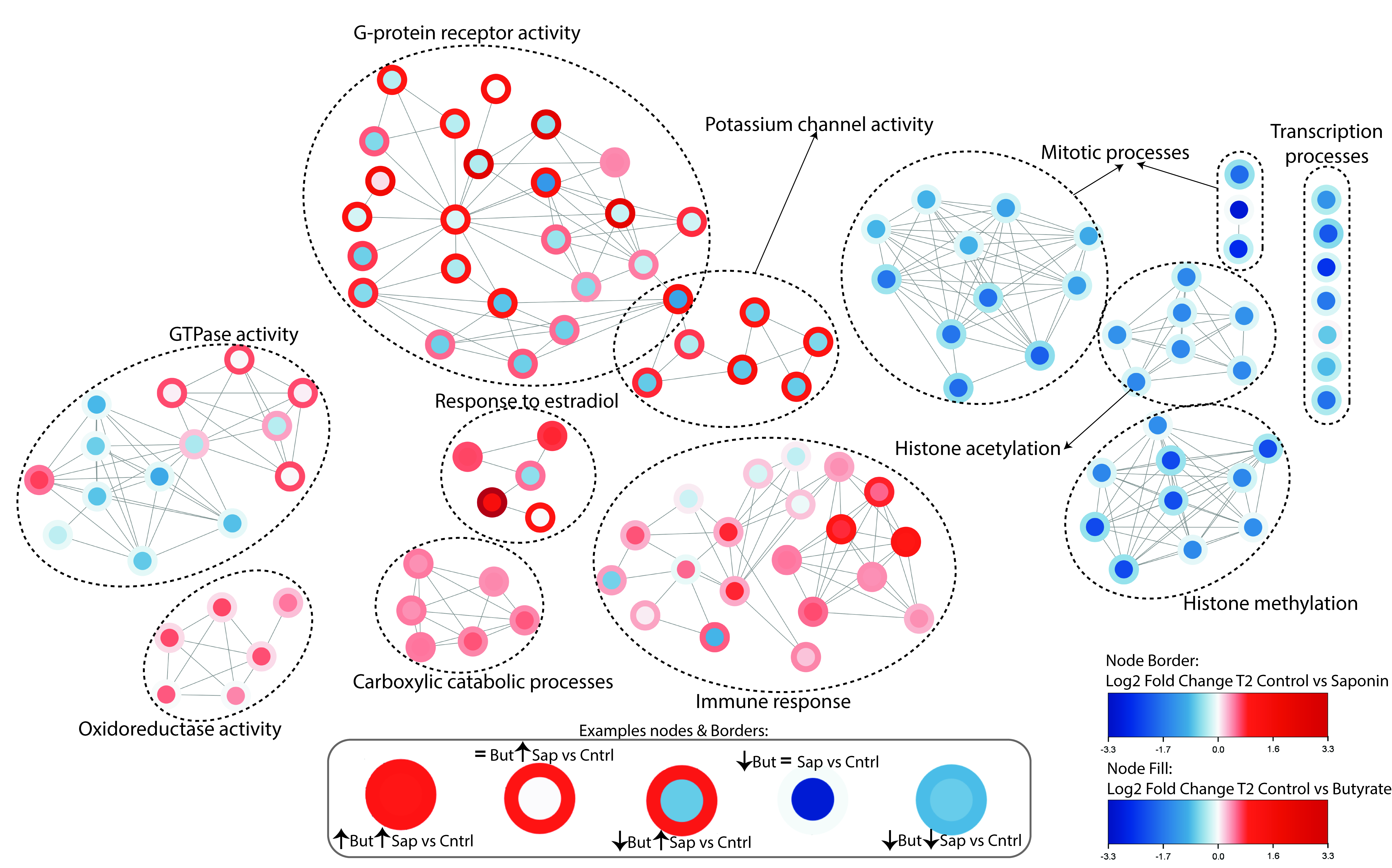

Supplement: Supplementary file 9 — Additional file 9: Gene expression of transcripts from zebrafish gut fed either a control, butyrate- or saponin-supplemented diets at 54 and 69 dpf. [file 42523_2023_230_MOESM9_ESM.zip › Supplementary folder 1/Untitled-1.tif]
